# Advances in silver nanoparticles: a comprehensive review on their potential as antimicrobial agents and their mechanisms of action elucidated by proteomics

**DOI:** 10.3389/fmicb.2024.1440065

**Published:** 2024-07-31

**Authors:** Adriana S. Rodrigues, Jorge G. S. Batista, Murilo Á. V. Rodrigues, Velaphi C. Thipe, Luciene A. R. Minarini, Patricia S. Lopes, Ademar B. Lugão

**Affiliations:** ^1^Institute for Energy and Nuclear Research, National Nuclear Energy Commission-IPEN/CNEN-SP, São Paulo, Brazil; ^2^Department of Radiology, School of Medicine, University of Missouri, Columbia, MO, United States; ^3^Federal University of São Paulo, Institute of Environmental, Chemical and Pharmaceutical Sciences, São Paulo, Brazil

**Keywords:** nanomaterials, silver nanoparticles, antimicrobial, antibiofilm, antimicrobial resistance, mechanism of action, proteomic analysis, protein expression

## Abstract

Nanoparticles play a crucial role in the field of nanotechnology, offering different properties due to their surface area attributed to their small size. Among them, silver nanoparticles (AgNPs) have attracted significant attention due to their antimicrobial properties, with applications that date back from ancient medicinal practices to contemporary commercial products containing ions or silver nanoparticles. AgNPs possess broad-spectrum biocidal potential against bacteria, fungi, viruses, and Mycobacterium, in addition to exhibiting synergistic effects when combined with certain antibiotics. The mechanisms underlying its antimicrobial action include the generation of oxygen-reactive species, damage to DNA, rupture of bacterial cell membranes and inhibition of protein synthesis. Recent studies have highlighted the effectiveness of AgNPs against various clinically relevant bacterial strains through their potential to combat antibiotic-resistant pathogens. This review investigates the proteomic mechanisms by which AgNPs exert their antimicrobial effects, with a special focus on their activity against planktonic bacteria and in biofilms. Furthermore, it discusses the biomedical applications of AgNPs and their potential non-preparation of antibiotic formulations, also addressing the issue of resistance to antibiotics.

## 1 Introduction

Nanomaterials are structures with dimensions between 1 and 100 nanometers, encompassing the field of nanotechnology (Husain et al., 2023). They include nanostructures, nanocomposites, nanofibers, and nanofilms, and can be composed of polymers, biomaterials, ceramics, metals, among others. They encompass a variety of structures with different morphologies, such as rods, tubes, films, and particles. Nanoparticles, a subset of nanomaterials, are specifically characterized by their three-dimensional nanoscale size and generally have spherical or quasi-spherical shapes. Their unique properties, such as high reactivity and large surface area, make them particularly suitable for applications in drug delivery, imaging, and catalysis (Khan et al., 2019; Cheng et al., 2023).

Obtaining nanoparticles can be carried out by chemical, physical and biological methods, often involving high costs and the use of toxic substances. On the other hand, synthesis using natural substances, such as phytochemicals, has been widely discussed due to their reducing potential, biocompatibility and low environmental toxicity. Green nanotechnology, based on the principles of green chemistry, seeks economic, social, health and environmental benefits by prioritizing the reduction of the generation of hazardous chemical waste and promoting safer applications (Nasrollahzadeh et al., 2019; Brar et al., 2022).

Analytical techniques for the physicochemical characterization of nanoparticles are fundamental for the detailed investigation of their properties, including structure, crystallinity, morphology, size, and surface chemistry. UV–Visible spectroscopy is used for the primary characterization of colloidal suspension particles, confirming the formation of NPs by the intensity of the surface plasmon resonance (SPR) band. The Dynamic Light Scattering (DLS) technique allows the determination of the hydrodynamic particle size, influenced by Brownian motion as a function of the diffusion coefficient, analyzing fluctuations in the intensity of scattered light to determine particle diffusion and directly relate it to size. The relationship between the diffusion coefficient and size is variable for spherical particles, as the diffusion coefficient decreases as the hydrodynamic radius of the particles increases, as described by the Stokes-Einstein equation. Additionally, it provides the polydispersity index, indicating system homogeneity. Nanoparticle Tracking Analysis (NTA) individually tracks the movement of each particle, determining its size based on Brownian motion. The Zeta potential measures the surface charge and stability of colloidal suspensions, indicating the tendency of particles to aggregate or repel each other. Inductively Coupled Plasma Mass Spectrometry (ICP-MS) is used for multielemental investigation, quantifying silver uptake in different systems. X-ray Diffraction (XRD) provides information on the crystallinity level and molecular structure of nanoparticles. Fourier Transform Infrared Spectroscopy (FTIR) and Raman spectroscopy identify the surface chemical composition and investigate particle functionalization, vibrational, and rotational modes. X-ray Photoelectron Spectroscopy (XPS) analyzes the surface chemistry of NPs, while Energy Dispersive X-ray Spectroscopy (EDX) identifies and quantifies the elemental composition of materials. Nuclear Magnetic Resonance (NMR) spectroscopy is used to identify and quantify surface functional groups. Imaging techniques, such as Scanning Electron Microscopy (SEM) and Transmission Electron Microscopy (TEM), provide detailed images of particle morphology and size, allowing the creation of size distribution histograms through statistical analysis. Atomic Force Microscopy (AFM) allows for the analysis of aggregation state, dispersion, topography, structure, shape, and size of nanoparticles. Together, these techniques provide comprehensive data on the structural, chemical, and morphological properties of nanoparticles, enabling an in-depth investigation of their characteristics (Pryshchepa et al., 2020; Patil and Chougale, 2021; Joudeh and Linke, 2022).

Silver has a long history as an antimicrobial agent and has been used since ancient times, with documented evidence in several medicinal applications. In 1881, Credé’s method introduced the use of a solution containing 2% AgNO_3_ to treat neonatal conjunctivitis. In the 19^th^ century, doctors used silver thread to suture surgical wounds, while during the First World War, silver foil was applied to soldiers’ wounds to prevent infection and aid wound healing. By the early 20^th^ century, colloidal silver became popular in hospitals as a germicidal agent. Additionally, silver salts were used to treat various infections, such as conjunctivitis, gastroenteritis, gonorrhea and syphilis (Medici et al., 2019; Kaiser et al., 2023).

Silver nanoparticles (AgNPs) are widely described in the literature as having a broad biocidal spectrum, covering Gram-positive and Gram-negative bacteria, fungi, viruses and mycobacteria, in addition to acting synergistically with antibiotics to enhance their effectiveness (Misirli et al., 2021). These properties are based on diverse and complex mechanisms, including the generation of reactive oxygen species (ROS), DNA damage, impairment of bacterial cell membranes and inhibition of protein synthesis (Dakal et al., 2016; Salleh et al., 2020; Bruna et al., 2021).

Recent studies have highlighted the effectiveness of AgNPs against a variety of bacterial strains, including clinical isolates and standard strains (ATCCs, NCTCs, PCCs, MTCCs) of various species, such as *Acinetobacter baumannii, Escherichia coli, Staphylococcus aureus, Staphylococcus epidermidis, Klebsiella pneumoniae, Pseudomonas aeruginosa, Pseudomonas fluorescens, Salmonella typhi, Enterococcus faecalis, Enterobacter* spp.*, Bacillus subtilis, Micrococcus luteus, Proteus vulgari*, among others (Shaik et al., 2018; Wang H. et al., 2021; Wang L. et al., 2021; Khane et al., 2022; Basheer et al., 2023; Kaiser et al., 2023). Furthermore, the synergy between AgNPs and antibiotics, such as penicillin G, amoxicillin, erythromycin, clindamycin, vancomycin, piperacillin, ciprofloxacin, tetracycline, levofloxacin, cefixime, cefazolin, neomycin, rifampicin, gentamicin, chloramphenicol, imipenem; has been shown to increase the effectiveness of these medications (Singh et al., 2013; Mazur et al., 2020; Aabed and Mohammed, 2021; Hassan et al., 2021; Haji et al., 2022).

This review addresses the mechanisms of action of AgNPs against planktonic and biofilm bacteria at the proteomic level, as well as their biomedical applications, aiming to enhance formulations and investigate synergism with antibiotics, highlighting advances relevant to nanomedicine and public health.

## 2 Biological interaction and compatibility of AgNPs

The diverse applications and the increase of AgNPs in the marketplace makes them inevitably present in the environment, as they are released not only during the use of products such as cosmetics, wound dressings, textiles, food packaging and personal care products, but also through their productive processes. This fact entails the ingestion of this NPs by humans by food or drinks that were in direct contact with these NPs or through contaminated by water containing these nanostructures, and their intake can also occur via dermal contact, inhalation or intravenous or intraperitoneal injections (Jaswal and Gupta, 2021; Zhang et al., 2022; Ren et al., 2023).

Once inside the body AgNPs are distributed through the organs and tissues, firstly accumulating in the lungs when entering via the respiratory tract. These nanoparticles can penetrate both healthy or injured skin when present in formulations for topical application such as cosmetics and wound dressings or even textiles and then enter circulatory system. Toxicity and pathological symptoms of AgNPs intake are modulated by some properties of these nanostructures, such as size, stability and aggregation condition. Zhang et al. (2022) summarized their toxic effects on different animal models, with different organs being affected.

Juling et al. (2016) administered commercial AgNPs (15–30 nm) coated with polyoxyethylene glycerol trioleate and Tween 20 (polyoxyethylene (20) sorbitan monolaurate) to rats via oral or intravenous injection and after 24 h measured their content on different tissues and feces. For the orally administered group, AgNPs accumulated in feces and colon, while intravenous administration resulted in a more distributed accumulation, being present mainly in lungs, then in feces, followed by the colon, intestine, kidney, testis and thymus, with an expected silver ions content below 15%. Juling et al. (2016) still also indicate the reduction in AgNPs size to 5 to 12 nm and the formation of silver acetate NPs of 6 nm in animals treated with silver ions (AgNO_3_).

Since the gastrointestinal tract is one of the main entry ways for many invaders, as pathogens and toxins, including AgNPs, Ren et al. (2023) evaluated the intestinal toxicity of these NPs *in vivo*. Mice were exposed daily for 4 weeks with 10 mL/kg body weight of commercial suspensions of AgNPs capped with poly(vinylpyrrolidone) of 5 mg/mL through oral gavage were compared to animals being treated with other NPs or water. The authors indicated severe damage for the animals treated with AgNPs including damage in the epithelia structure, reduction in the mucosa layer thickness and alterations in the local microbiota. Additionally, the interaction of immune cells with these NPs resulted in the generation of reactive oxygen species (ROS) that induced uncontrolled apoptosis of dendritic cells, affecting the normality of the immune microenvironment in the intestine of these animals. Among the tested NPs (Ag, TiO_2_, SiO_2_, and ZnO) silver was the most harmful.

The cytotoxicity of AgNPs is generally related to the production of large amounts of ROS, affecting cytoskeleton, DNA, DNA repair enzymes and inducing cell apoptosis. However, these effects are associated with size and the synthetic methods employed. Larger NPs present less cytotoxicity due to the reduction in surface area and, consequently, the reduction in interaction with cell membranes and organelles (Jaswal and Gupta, 2021; Zhang et al., 2022). Amr et al. (2023) biosynthesized AgNPs employing aqueous extract of a mushroom (*Agaricus bisporus*) and evaluated their cytotoxicity against human skin fibroblasts, obtaining cell viability of 98.2% for the diluted solutions (0.04 μg/mL) until 82.3% for 25.0 μg/mL samples. Besides the dose-dependent cytotoxicity, it is worth pointing out that the size of the NPs was 766 nm, and the use of safer aqueous extracts as reducing agents instead of commonly applied chemicals for this purpose. This approach eliminates purification steps to reduce the presence of these compounds that tend to increase cytotoxicity.

Yadav and Preet (2023) compared AgNPs prepared by chemical (sodium borohydride) and green (clove bud extract) methods of synthesis and observed a better larvicidal (*Aedes aegypti*) effect with lower toxicity (*Daphnia magna*) for the samples prepared with plant extract. Despite the recognized toxicity of AgNPs some approaches can reduce this drawback, such as the use of natural reducing and capping agents, the control of size and the use of these NPs in combination to other antimicrobial agents to attempt microbial control through different and complementary mechanisms, reducing the necessary concentration of AgNPs.

## 3 Applications of silver nanoparticles as antimicrobial agents

Nanomaterials are structures that exist on the nanometer scale (10^-9^). According to the American Society for Testing and Materials (ASTM), nanoparticles are a specific category of particles with lengths in two or more dimensions within the range of 1 to 100 nm (Loiseau et al., 2019).

The characteristics of these metallic nanoparticles depend on factors such as size, morphology, stability, surface charge, and other physical and chemical properties. These properties can be influenced through the kinetic interaction conditions between metal ions and reducing agents, by modifying synthesis methods, characteristics of the reducing agents, and the use of stabilizers. Additionally, various morphologies of AgNPs can be obtained, such as cubic shapes, spherical, nanorods, nanowires, nanobars, triangular and pyramidal, in a controlled manner by modifying thermodynamics and kinetics during synthesis, through factors such as the type of reducer (chemical or physical), use of solvent systems, variations in temperature, pH, pressure, and controlled atmosphere (Zhang et al., 2016; Burdușel et al., 2018).

The preference for biological methods in the synthesis of AgNPs has driven significant advances in the search for more ecological and efficient processes. This approach is based on the use of bacteria, plant extracts, and biomolecules as non-toxic reducing agents, offering a safe, environmentally friendly, and economically advantageous alternative for AgNPs production. Biological methods are distinguished by their safer and less harmful approach, supported by green nanotechnology stemming from ‘green chemistry’, aiming for beneficial innovations for health, the environment, and industry, ensuring efficiency and safety in meeting current needs with greater sustainability (Salleh et al., 2020; Bamal et al., 2021).

AgNPs can be used in different sectors, such as medicine, engineering, agriculture, and the environment sciences (Dawadi et al., 2021). Major applications include surface coating and medical device coating, advanced dressings, theranostics, cosmetics, textiles and electronics, sensors, and catalysts (Prabhu and Poulose, 2012). Their properties include antibacterial, antifungal, antiviral, anti-inflammatory, anti-angiogenic, and antitumor activities (Pryshchepa et al., 2020).

Bacterial resistance to antibiotics represents a global health challenge. The World Health Organization (WHO) warns that drug-resistant pathogens significantly contribute to high mortality rates (Bamal et al., 2021). Consequently, research and development of new antimicrobial agents have attracted considerable interest. Among the various known metallic nanoparticles, such as Au, Pd, Cu, and Zn, silver stands out as one of the most effective in combating pathogenic microorganisms. Studies often cite its broad-spectrum antimicrobial capacity due to its large surface area and multivalent interactions (Sánchez-López et al., 2020; Bruna et al., 2021).

Table 1 presents significant studies exploring the potential of AgNPs in various applications, such as antimicrobial, antifungal, and antiviral action. These studies address synthetic methods, morphology, size, and the different targets to which these nanoparticles are currently being applied.

**Table 1:** Silver nanoparticles and antimicrobial applications.

Nanoparticles	Description	Shape	Size	Species/Strain	Effect/Concentration	Method	References
AgNPs *of L. acapulcensis*	Green synthesis of chemically reduced AgNPs by *Lysiloma acapulcensis*	Spheric	1.2–62 nm	*C. albicans, E. coli, S. aureus*, and *P. aeruginosa*	Inactivation 0.06–0.25 μg/mL	Disk diffusion	Garibo et al. (2020)
AgNPs *of Citrus limon* (L.)	Green synthesis of chemically reduced AgNPs by aqueous extract of *Citrus limon* (L.) zest	Spheric and cubic	7–28 nm	*S. aureus, E. coli*, and *C. albicans*	Inactivation	Disk diffusion	Khane et al. (2022)
Sp-AgNPs	Green synthesis of AgNPs using aqueous root extract of *Salvadora persica* (Sp) as reducing agent	Spheric and rods	37.5 nm	*E. coli* (ATCC 11229) and *S. epidermidis* (ATCC 12228)	Inactivation 0.39–0.78 μg/mL and 0.19–0.39 μg/mL	Disk diffusion	Arshad et al. (2021)
AgNPs of *Cynodon dactylon*	Green synthesis of AgNPs reduced by *Cynodon dactylon* leaf extract	Spheric	15 nm	*P. fluorescens*	Inactivation	Disk diffusion	Wang et al. (2021)
MOF-AgNPs	Green synthesis of AgNPs reduced by *Moringa oleifera* flower extract	Spheric	22 nm	*K. pneumoniae* and *S. aureus*	Inactivation	Disk diffusion	Bindhu et al. (2020)
*Sb*-AgNP	Green synthesis of reduced AgNPs with aqueous extract of *Scutellaria barbata*	Spheric	20–40 nm	*E. coli, P. aeruginosa, S. aureus* and *K. pneumoniae*	Inactivation 2.8, 3.1, 3.4, and 2.2 μL	Disk diffusion	Veeraraghavan et al. (2021)
AgNPs of *Phyllanthus emblica*	Biological synthesis of AgNPs with *Phyllanthus emblica* fruit extract	Spheric	19–45 nm	*K. pneumoniae* and *S. aureus*	Inactivation 10 μg both	Disk diffusion	Renuka et al. (2020)
AgNPs of *Gardenia thailandica* (GTLE)	Green synthesis of AgNPs reduced by *Gardenia thailandica* leaf extract (GTLE)	Spheric	11.02–17.92 nm	*S. aureus*	Reduction of CFU, regeneration of the epidermis and reduction of inflammatory cell infiltration 4-64 μg/mL	Disk diffusion and *in vivo* antibacterial activity in rats	Attallah et al. (2022)
AgNPs of *Penicillium oxalicum*	Biogenic synthesis of AgNPs from fungal metabolites of *Penicillium oxalicum*	Spheric	60–80 nm	*S. aureus, S. dysenteriae* and *S. typhi*	Inactivation	Disk diffusion and broth dilution	Feroze et al. (2020)
*OV*-AgNPs	Green synthesis of AgNPs reduced and stabilized with plant extract of *Origanum vulgare* L.	Spheric	2–25 nm	*E. coli, P. aeruginosa, S. typhi, S. sonnei, M. luteus, S. epidermidis, S. aureus methicillin-resistant (MRSA), S. aureus, A. flavus, A. suplente, P. alba* and *P.* var*iotii*	Inactivation	Disk diffusion	Shaik et al. (2018)
AgNPs of *Berberis vulgaris*	Green synthesis of reduced AgNPs with aqueous extract of *Berberis vulgaris* leaves and roots	Spheric	30–70 nm	*E. coli* and *S. aureus*	Inactivation or reduction 0.20 and 400 μg/mL	Disk diffusion and broth dilution	Behravan et al. (2019)
AgNPs of *Padina* sp.	Green synthesis of AgNPs reduced by aqueous extract of marine macroalgae *Padina* sp.	Spheric	25–60 nm	*S. aureus, B. subtilis, P. aeruginosa, S. typhi* and *E. coli*	Inactivation 0.25 mg/mL for all strains	Disk diffusion	Bhuyar et al. (2020)
AgNPs of *Carissa carandas L.*	Green biosynthesis of silver nanoparticles using *Carissa carandas* L leaf extract.	Not specified	Not specified	*S. flexneri, Citrobacter* spp.*, S. typhimurium, E. faecalis,* and *Gonococos* spp.	Inactivation 60, 80, and 100 μL for other strains	Disk diffusion	Singh et al. (2021)
AgNPs of *Cymbopogon citratus*	Biosynthesis of AgNPs with *Cymbopogon citratus* leaf extract	Not specified	Estimated of 47 nm	*S. typhi, B. cereus and S. flexneri*	Inactivation 150 μg/mL and 50 μg/mL for other strains	Disk diffusion and broth dilution	Rakib-Uz-Zaman et al. (2022)
AgNPs of *Cynara scolymus* L.	Green synthesis of AgNPs reduced by artichoke waste extract *Cynara scolymus* L.	Spheric	28.78 nm	*S. aureus* (ATCC 25923), *E. coli* (ATCC 25922), *C. albicans, B. subtilis* (ATCC 11774) and *P. aeruginosa* (ATCC 27853)	Inactivation 0.12, 0.13, 0.03, 0.25, and 0.07 μg/mL	Broth dilution	Baran et al. (2021)
AgNPs of *Talaromyces purpureogenus*	Green synthesis of AgNPs using fungus *Talaromyces purpureogenus* isolated from *Taxus baccata Linn.*	Spheric	30–60 nm	*E. coli, S. typhi, L. monocytogenes and S. dysenteriae*	Inactivation 0.78125, 3.125 μg/mL and 1.5625 μg/mL for other strains	Disk diffusion	Sharma et al. (2022)
AgNPs of *Aloe vera*	Green synthesis of AgNPs from *Aloe vera* leaf extract	Not specified	Not specified	*E. coli, P. aeruginosa, Enterobacter* spp. and *S. aureus*	Inactivation	Disk diffusion	Anju et al. (2020)
AgNPs of Phingobium sp. MAH-11	Biological synthesis of AgNPs using *Phingobium* sp. MAH-11	Spheric	7–22 nm	*S. aureus* and *E. coli*	Inactivation 6.25 and 50 μg/mL	Disk diffusion and broth dilution	Akter and Huq (2020)
AgNPs of *Acacia cyanophylla*	Green synthesis of AgNPs using aqueous extract of *Acacia cyanophylla*	Spheric	88.11 nm	*E. coli*	Inactivation 3.125–12.5 μg/mL	Broth dilution	Jalab et al. (2021)
AgNPs of *Carthamus tinctorius* L.	Green synthesis of AgNPs using safflower (*Carthamus tinctorius* L.) waste extract	Spheric	8.67 nm	*S. aureus* and *P. fluorescens*	Inactivation or reduction 1.9 and 7.8 μg/mL	Disk diffusion and broth dilution	Rodríguez-Félix et al. (2021)
Ag-NC	Nanocomposition loaded with Ag-NPs by green methodology stabilized by polysaccharides	Spheric	15 nm	*E. coli* (ATCC 25922), *P. aeruginosa* (ATCC 27853), *S. aureus* (ATCC 23235) and *B. subtilis* (ATCC 23857)	Inactivation 20 and 40 μg/mL for other bacterial strains	Disk diffusion	Hasanin et al. (2021)
AgNPs of *Nigella sativa* and *Piper nigrum* L.	Green synthesis of silver nanoparticles using aqueous extract of *Nigella sativa* and *Piper nigrum L*	Spheric	20–50 nm	*B. megaterium, B. subtilis* (SK09), *S. aureus* (ATCC 6538), *E. coli* (ATCC 11775), *K. oxytoca* and *P. aeruginosa* (ATCC 27853)	Inactivation	Disk diffusion	Mahfouz et al. (2020)
AgNPs of *Bauhinia tomentosa Linn*	AgNPs biosynthesized from *Bauhinia tomentosa Linn*	Spheric	32 nm	*E. coli* (MTCC 732) and *S. aureus* (MTCC 3160)	Inactivation	Disk diffusion	Renganathan et al. (2021)
AgNPs of *Anagallis monelli*	AgNPs biosynthesized using *Anagallis monelli*	Spheric	20 nm	*E. coli, K. pneumoniae, S. marcescens, S. aureus* and *M. luteus*	Inactivation 4, 2, 8, 16, and 16 mg/mL	Disk diffusion and broth dilution	Dridi et al. (2022)
AgNPs of *Citrus limetta*	Green synthesis of AgNPs using *Citrus limetta* peel extract	Spheric	18 nm	*M. luteus, S. mutans, S. epidermidis, S. aureus* and *E. coli.*	Inactivation and antibiofilm 4.75 μg/mL to all strains	Disk diffusion and broth dilution	Dutta et al. (2020)
AgNPs	Biosynthesis of silver nanoparticles using marine fungi *Penicillium simplicissimum, Aspergillus terreus, Aspergillus japonicus* and *Aspergillus oryzae*	Spheric	3.8–23 nm	*E. coli, K. pneumoniae, P. vulgaris, S. typhi, E. faecalis, S. aureus* methicillin resistant (MRSA), *S. hominis* and *S. epidermidis*	Inactivation	Agar well diffusion	Basheer et al. (2023)
AgNPs of *Bacillus subtilis*	Biosynthesis of AgNPs using isolated *Bacillus subtilis*	Spheric	20 nm	*E. coli, S. aureus, P. aeruginosa, B. cereus* and S*. typhi*	Inactivation 42.1, 42.5, 169.3, 43.2, and 42.8 μg/mL	Broth dilution	El-Bendary et al. (2020)
ML-AgNPs	Green synthesis of AgNPs using *Morinda lucida* leaf extract	Spheric	11 nm	*Citrobacter, E. coli, P. vulgaris, S. typhi, V. cholerae* and *E. faecalis*	Inactivation	Disk diffusion	Labulo et al. (2022)
AgNPs of *Myrsine africana*	Green synthesis of AgNPs using *Myrsine africana* leaf extract	Spheric	28.32 nm	*P. aeruginosa, S. aureus, E. coli, K. pneumoniae* and *P. mirabilis*	Inactivation or reduction 0.03 mg/mL for all strains	Agar well diffusion	Sarwer et al. (2022)
AgNPs of *Syzygium cumini*	Green synthesis of AgNPs using fruits extracts of *Syzygium cumini*	Almost spheric	47 nm	*S. aureus, B. subtilis, P. aueruginosa* and *E. coli*	Inactivation 25 μg/mL for all strains	Disk diffusion	Chakravarty et al. (2022)
AgNPs of *Gelidium corneum*	Green synthesis of AgNPs using marine red algae *Gelidium corneum*	Spheric	20–50 nm	*E. coli*	Inactivation 0.26 μg/mL	Broth dilution	Yılmaz Öztürk et al. (2020)
AgNPs of *Hypericum perforatum L.*	Green synthesis of AgNPs using *Hypericum perforatum* L. aqueous extract	Spheric	20–40 nm	*P. aeruginosa* (ATCC 13048), *K. pneumoniae β-lactamase, E. coli specific spectrum β-lactamase* (ESBL), *E. coli* (ATCC 25922), *S. aureus* (ATCC 43300), *B. cereus* (ATCC 11778), and *B. subtilis* (ATCC 6633)	Inactivation or reduction 6.25, 12.5 μg/mL, n/d, n/d, 12.5, 6.25, and 12.5 μg/mL	Disk diffusion, broth dilution and growth curve	Alahmad et al. (2022)
OE-Ag	Green synthesis of AgNPs using *Olea europaea* leaf extract	Spheric	8 nm	*P. aeruginosa* (ATCC 27853TM), *K. pneumonia* (ATCC 13883), *S. aureus* (ATCC 15564) and *B. subtilis* (ATCC 6051)	Reduction of CFU	Broth dilution	Sellami et al. (2021)
GT AgNPs	Green synthesis of silver nanoparticles using green tea leaf extract	Spheric	15–33 nm	*S. aureus* and *Klebsiella* sp.	Inactivation 5 mg/mL for both	Disk diffusion	Widatalla et al. (2022)
AgNPs-LCg and AgNPs-FCg	Green synthesis of AgNPs using *Calotropis gigantea* leaf and flower	Spheric	Hydrodynamic size of 163.5–256.7 and 188.35–227.65 nm	*E. coli* and *S. aureus*	Inactivation	Disk diffusion	Kemala et al. (2022)
Chi/Ag-NPs	AgNPs stabilized with chitosan	Almost spheric	9–65 nm	*S. aureus and P. aeruginosa*	Inactivation 12.5 μg/mL for both	Disk diffusion and broth dilution	Shehabeldine et al. (2022)
AgNP-S, AgNP-F and AgNP-W	AgNPs biosynthesized from *Carduus crispus*	Not specified	131, 33, and 70 nm	*E. coli* and *M. luteus*	Inactivation	Disk diffusion	Urnukhsaikhan et al. (2021)
GCL·AgNPs	AgNPs phytosynthesized from *Glochidion candolleanum* leaves	Spheric and ellipsoidal	Not specified	*B. subtilis* (ATCC 6633), *L. monocytogens* (ATCC 19115), *S. aureus* (ATCC 6538), *E. coli* (ATCC 8739), *P. aeruginosa* (ATCC 9027) and *S. enterica* (ATCC 14028)	Inactivation	Disk diffusion	Balachandar et al. (2022)
SA-AgNPs, GL-AgNPs and BR-AgNPs	Biocompatible AgNPs (AgNPs) from leaf extracts of *Semecarpus anacardium, Glochidion lanceolarium* and *Bridelia retusa*	Spheric	62.72, 93.23, and 74.56 nm	*P. aeruginosa* (MTCC 741), *E. coli* (MTCC 739) and *S. aureus* (MTCC 96)	Inactivation	Broth dilution	Mohanta et al. (2020)
AgNPs of *Cestrum nocturnum*	AgNPs synthesized by *Cestrum nocturnum*	Spheric	20 nm	*Citrobacter, E. faecalis, S. typhi, E. coli, P. vulgaris* and *V. cholerae*	Inactivation 16, 4, 16, 16, 8, and 8 μg/mL	Disk diffusion and broth dilution	Keshari et al. (2020)
ZZAE-Ag-NPs and ZZEE-Ag-NPs	Synthesis of AgNPs from extracts of Wild Ginger (*Zingiber zerumbet*)	Spheric	Hydrodinamic size 24.28–153.2 nm	*S. aureus, E. faecalis* and *E. mutans*	Inactivation ZZAE-Ag-NPs: 25, 6.5, and 25 μg/mL	Disk diffusion	Ramzan et al. (2022)
					ZZEE-Ag-NPs: 3.12, 6.25, and 12.5 μg/mL		
AgNPs/EML, AgNPs/EMF, AgNPs/EMDS, AgNPs/EML, AgNPs/EMF and AgNPs/EMDS	Green synthesis of AgNPs *Morinda citrifolia* L. (noni)	Spheric	3–11 nm	*E. coli* and *S. aureus*	Inactivation	Disk diffusion	Morales-Lozoya et al. (2021)
AgNPs of *Sapindus mukorossi*	Green synthesis of AgNPs using *Sapindus mukorossi* fruit pericarp extract	Spheric	17.3 nm	*P. aeruginosa* (ATCC 27853) and *S. aureus* (ATCC 25923)	Inactivation 15 μg/mL for both	Disk diffusion	Huong, and V., and Nguyen, N. T. (2019)
AgNPs of *Rubus ellipticus Sm.*	Green Synthesis of AgNPs from root extracts of *Rubus ellipticus* Sm.	Spheric	13.85–34.30 nm	*E. coli, S. aureus, K. pneumoniae* and *E. faecalis*	Inactivation	Disk diffusion	Khanal et al. (2022)
AgNps of *Shewanella* sp. ARY1	Biosynthesis of AgNPs using culture supernatant of *Shewanella* sp. ARY1	Spheric	38 nm	*E. coli* and *K. pneumoniae*	Inactivation 8–16 μg/mL	Disk diffusion and broth dilution	Mondal et al. (2020)
AgNps of *Trigonella foenum-graecum*	Biogenic synthesis of AgNPs using *Trigonella foenum-graecum* seed extract	Spheric	82.53 nm	*E. coli* (ATCC 25922), *S. aureus* (ATCC 25923) and *B. cereus* (ATCC 11778)	Inactivation	Disk diffusion	Awad et al. (2021)
CSE-AgNPs and PAE-AgNPs	AgNPs using *Camellia sinensis* leaf extract (CSE) and *Prunus africana* bark extract (PAE)	Spherical and aggregated in layers	CSE-AgNPs 3–98 nm and PAE-AgNPs 4–94 nm	*E. coli* (ATCC 96522) and *K. pneumoniae* (NTCT 9633)	Inactivation CSE-AgNPs: 0.125 and 0.25 mg/mL	Disk diffusion and broth dilution	Ssekatawa et al. (2021)
					PAE-AgNPs: 0.125 and 0.25 mg/mL		
AgNPs of *Punica granatum*	Green synthesis of AgNPs using the aqueous extract of *Punica granatum* bark	Spheric	20–40 nm	*E. coli* (ATCC 25922), *P. aeruginosa* (ATCC 27584)*, P. vulgaris* (ATCC 8427), *S. typhi* (ATCC 14028), *S. aureus* (ATCC 29213), *S. epidermidis* (MTCC 3615) and *K. pneumoniae*	Inactivation	Disk diffusion and broth dilution	Devanesan et al. (2018)
AgNPs of *Azadirachta indica*	AgNPs reduced by *Azadirachta indica* extract	Spheric	65 nm	*P. aeruginosa*	Inactivation 2 μg/mL	Disk diffusion	Senthilkumar et al. (2018)
OLAgNPs	Green biogenic of AgNPs using polyphenolic extract of olive leaf wastes	Spheric	20–45 nm	*L. monocytogenes, B. cereus, S. aureus, E. coli, Y. enterocolitica* and *C. jejuni*	Inactivation 5, 5, 5, 5, 25, and 25 μg/mL	Disk diffusion	Alowaiesh et al. (2023)
AgNPs-BM and AgNPs-WM	Biogenic AgNPs of Agaricus bisporus from white mushroom extract and brown mushroom extract	Spheric	5 nm AgNPs-BM and 11 nm AgNPs-WM	*S. aureus, S. epidermis, B. subtilis, E. coli, S. typhi* and *P. aeruginosa*	Inactivation	Disk diffusion	Al-Dbass et al. (2022)
MOAgNPs	Green biogenic AgNPs using aqueous extract of *Moringa Oleifera*	Spheric	5–50 nm	*E. coli, S. marcescens, S. aureus* and *B. subtilis*	Inactivation 5.70, 4.10, 3.15, and 2.75 μg/mL	Disk diffusion	Abdel-Rahman et al. (2022)
EC-AgNPs and TA-AgNPs	Green AgNPs using extracts from *Eucalyptus camaldulensis* and Terminalia arjuna	Spheric	100 nm EC-AgNPs and 35 nm TA-AgNPs	*B. subtilis, S. aureus, E. coli*, and *P. multocida*	Inactivation	Disk diffusion	Liaqat et al. (2022)
bAgNPs	Biogenic AgNPs synthesized using Syzigyum cymosum extract	Spheric	17.2-35.3 nm	*B. subtilis, E. coli* DH5α*, E. coli* K12, enteropathogenic *E. coli* and *Salmonella typhi*	Inactivation 0.125 μg/mL for all strains	Disk diffusion and broth dilution	Mahmud et al. (2022)
AgNPs	Green synthesis using natural reducing agents present in extracts of apple, orange, potato, red pepper, white onion, garlic and radish	Spheric	9-30 nm	*S. aureus* ATCC 6538, *B. cereus* ATCC 10987 and *E. coli* ATCC 11229	Inactivation AgNPs + Potato: 0.016, 0.004, 0.016 μg/mL	Broth dilution	Wasilewska et al. (2022)
					AgNPs + Garlic: 2.641, 0.066, 0.066 μg/mL		
					AgNPs + White onion: 0.066, 0.016, 2.641 μg/mL		
					AgNPs + Radish: 42.250 μg/mL		
					AgNPs + Red pepper: 10.563, 2.641, 10.563 μg/mL		
					AgNPs + Orange: 10.563, 0.066 μg/mL		
					AgNPs + Apple: 0.066, 0.016, 2.641 μg/mL		
AgNPs-KP	Green synthesis of AgNPs from *Klebsiella pneumoniae* (AgNPs-KP)	-	38.9 nm	*K. pneumoniae* carbapenemase (KpC)	9.76 μg/mL	Broth dilution	Chuy et al. (2022)
SX-AgNPs	Green synthesis of *Solanum xanthocarpum* fruit capped silver nanoparticles	Spheric	22.45 nm	*E. coli, Shigella* spp.*, P. aeruginosa* and *Aeronomas* spp.	Inactivation 2.5, 2.5, 1.25, and 1.25 mg/mL	Broth dilution	Pungle et al. (2021)
AN-AgNPs	Green synthesis of AgNPs using *Argyreia nervosa* leaf extract	-	10–40 nm	Enteropathogenic *E. coli* (EPEC)	Inactivation	Disk diffusion	Parvathalu et al. (2023)
B-AgNPs, L-AgNPs and LB-AgNPs	AgNPs biosynthesis using mixture of *Lactobacillus* sp. and *Bacillus* sp. growth	Spheric	B-AgNPs 11–22.8 nm, L-AgNPs 7.97–14.3 nm and LB-AgNPs 4.65–11.3 nm	*P. aeruginosa* and *S. aureus*	Inactivation 20 μg/mL for B-AgNPs in *P. aeruginosa* and 10 μg/mL for the others AgNPs and bacteria strains	Disk diffusion	Al-asbahi et al. (2024)
AgNPs of Dsr1KO, Dsr9KD and Dsr20KD	AgNPs biosynthesized from the sRNA deletion of strains *D. radiodurans* (Dsr1KO, Dsr9KD e Dsr20KD)	Spheric	10–20 nm	*P. aeruginosa, E. coli* and *S. epidermidis*	Reduction and inactivation AgNPs Dsr1KO, Dsr9KD e Dsr20KD > 12 μg/mL for *P. aeruginosa* and *E. coli* and > 90 μg/mL for *S. epidermidis*	Broth dilution	Chen et al. (2021)
AgNPs	Biosynthesis of AgNPs from the *Nocardiopsis* sp. -MW279108	Spheric	2.6–10 nm	*B. subtilis, B. cereus, A. baumannii, E. coli, P. aeruginosa, S. typhimorium* and *S. aureus*	Inactivation 214 μg/mL	Disk diffusion	Abada et al. (2021)
AgNPs	Green synthesis of AgNPs of secondary metabolites of *Bacillus subtilis* (SDUM301120)	Spheric	2–26 nm	*E. coli* ATCC 25922*, S. aureus* ATCC 29213*, V. parahemolyticus* ATCC 17802^T^ and *A. baumannii* ATCC 19606^T^	Inactivation 8.1, 8.3, 16.2, and 8 μg/mL	Disk diffusion	Yu et al. (2021)
Cp-AgNPs	Biosynthesis of AgNPs using *Cucumis prophetarum* aqueous leaf extract	Spheric	30–50 nm	*S. aureus* MTCC96 and *S. typhi* ATCC13076	Inactivation 20 μg/mL	Disk diffusion	Hemlata et al. (2020)
bAgNPs	Synthesis of biogenic AgNPs using *Caesalpinia digyna*	-	11.3–45.4 nm	*Bacillus subtilis, Escherichia coli* DH5α*, E. coli* K12, Enteropathogenic *E. coli (EPEC)* and *Salmonella typhi*	Inactivation 0.125 μg/mL	Disk diffusion	Niloy et al. (2020)
*P.y*AgNPs	Biogenic AgNPs using *Pyropia yezoensis*	Spheric	20–22 nm	*P. aeruginosa*	Reduction and inactivation 200 e 400 μg/mL	Disk diffusion	Ulagesan et al. (2021)
AgNP-His	Biosynthesis of AgNPs using the *Lippia abyssinica* plant leaf extract.	Spheric	5–14 nm	*S. aureus* ATCC 25926 *and E. coli* ATCC 25922	Inactivation 62.5 μg/mL	Disk diffusion	Shumi et al. (2023)
L-AgNPs	Green synthesis of Lignin-capped AgNPs	Spheric	14.01 nm	*E. coli*	Inactivation 0.1 mg/mL	Disk diffusion	Cao et al. (2021)
Bio-AgNPs	Biosynthesis of AgNPs by marine actinobacterium *Nocardiopsis dassonvillei*	Spheric	29.28 nm	*S. aureus,* CoNs *Staphylococcus, P. aeruginosa,* ESBL-producing *E. coli, Salmonella* sp.*, K. pneumoniae* and *P. mirabilis*	Inactivation 128, 128, 4, 64, 32, 64 and 64 μg/mL	Broth dilution	Khalil et al. (2022)

*Aspergillus flavus, Aspergillus fumigatus, Aspergillus niger, Aspergillus terreus, Bacillus cereus, Bacillus megaterium, Bacillus subtilis, Candida albicans, Candida glabrata, Candida krusei, Candida parapsilosis, Candida sake, Candida tropicalis, Enterococcus faecalis, Escherichia coli, Klebsiella oxytoca, Klebsiella pneumoniae, Listeria monocytogenes, Micrococcus luteus, Paecilomyces variotii, Penicillium expansum, Pityriasis alba, Proteus mirabilis, Proteus vulgaris, Pseudomonas aeruginosa, Pseudomonas fluorescens, Rhizopus oryzae, Salmonella enterica, Salmonella typhi, Serratia marcescens, Shigella dysenteriae, Shigella flexneri, Shigella sonnei, Staphylococcus aureus, Serratia marcescens, Staphylococcus epidermidis, Staphylococcus hominis, Streptococcus mutan, Pasteurella multocid, Vibrio parahemolyticus*, and *Vibrio cholerae*.

The search for new antimicrobial agents has been driven by the increasing number of infections presenting bacterial resistance to conventional antibiotic treatment, a which poses significant challenge for global health. The indiscriminate use of antibiotics has led to the development of multidrug-resistant microorganisms and consequently antimicrobial resistance (MDR; Hamad et al., 2020; Salleh et al., 2020). This microbial resistance represents a serious health problem, potentially raising morbidity and mortality rates, especially in pandemic and epidemic diseases, as highlighted by the World Health Organization (WHO). The emergence of bacteria resistant to antibacterial agents underscores the need to development of more effective antimicrobial agents to overcome these resistance profiles (Das et al., 2020).

According to the WHO, pathogens identified as top-level are categorized as ESKAPE, namely: *Enterococcus faecium*, *Staphylococcus aureus*, *Klebsiella pneumoniae*, *Acinetobacter baumannii*, *Pseudomonas aeruginosa*, and Enterobacter species. These pathogens pose significant challenges to human health, as they are the most prevalent in infections caused by MDR. Gram-negative bacteria exhibit a more pronounced profile of microbial resistance compared to Gram-positive strains (Kaiser et al., 2023).

The use of nanoparticles represents a substantial advance in the development of new therapeutics to tackle microbial resistance. Several authors have demonstrated the ability of AgNPs to significantly reduce biofilm formation and bacterial adhesion (Agreles et al., 2022). There is also the possibility of employing NPs in synergy with traditional antibiotics. Due to the unique properties of these nanomaterials, they could potentially reduce the dose and toxicity of antibiotics optimizing treatment compared to the exclusive use of antibiotic (Lee et al., 2019; Kukushkina et al., 2021).

The advantages of using AgNPs lie in their ability to interact with different bacterial mechanisms by targeting multiple pathways, thereby potentially increasing the spectrum of antimicrobial action (Ribeiro et al., 2022). By interacting with a broad spectrum, they can interfere with metabolic pathways by inducing ROS formation, inhibiting and/or modifying enzymes and proteins, reducing cell permeability, and causing homeostatic imbalance (Lee et al., 2019; Agreles et al., 2022).

Several authors have demonstrated the importance of synergy between silver nanoparticles and antibiotics, as evidenced in Table 2. This table presents relevant information on the interaction of these NPs with different antibiotics, highlighting potential synergistic effects that can result in greater effectiveness in combating various strains of microorganisms, including resistant ones.

**Table 2:** Silver nanoparticles in synergism with antibiotics.

Nanoparticles	Shape	Size	Antibiotic	Species/Strain	Effect/Concentration	References
Green synthesis of AgNPs using *Syzygium aromaticum* ethanolic extract (SAEE) and functionalized with chitosan (CS-AgNPs)	Spheric	80–120 nm	Amoxicillin (AMX), cefixime (CEF) and levofloxacin (LVX)	*E. coli* (LT 01253), *K. pneumoniae* (LT 0471), *S. aureus* (LT 3512), *S. typhi* (LT 01057) and *P. aeruginosa* (LT 0261)	Significant reduction in MICs of all antibiotics when combined with CS-AgNPs against pathogenic strains with maximum reduction in AMX	Asghar et al. (2020)
					CS-AgNPs + AMX: 4/64, 8/32, 4/16, 4/16 and 4/32 μg/mL	
					CS-AgNPs + CEF: 4/32, 4/8, 4/16, 4/2 and 4/32 μg/mL	
					CS-AgNPs + LVX: 4/1, 8/4, 4/4, 2/0.0625 and 4/128 μg/mL	
Green synthesis of AgNPs using *Withania coagulans extract*	Spheric	10–40 nm	Levofloxacin (LVX)	*E. faecalis, S. aureus, E. coli, P. vulgaris, S. typhi* and *V. cholerae*	AgNPs-Levo demonstrate lower MIC value compared to AgNPs and Levofloxacin alone. Furthermore, the Drug Combination Index (FICI) values showed synergistic and additive behavior AgNPs+LVX: 0.25, 0.25, 0.5, 16, 0.25 and 8 μg/mL	Keshari et al. (2020)
Biosynthesis of AgNPs using *A. baumannii* strain	Spheric	1–9 nm	Imipenem (IMI), ceftriaxone (CRO), cefepime (FEP) and ceftazidime (CAZ)	*Klebsiella* sp., *P. aeruginosa, A. baumannii* and *Proteus* sp.	All antibacterial combinations with AgNPs demonstrated significant (*p* < 0.0001) synergistic (FIC ≤ 0.5) and partial synergistic (0.5 < FICI <1) effects against all tested bacteria	Haji et al. (2022)
					AgNPs+IMI: 0.56, 0.56, 0.5 and 0.37 μg/mL	
					AgNPs+CRO: 0.26, 0.26, 0.5 and 0.51 μg/mL	
					AgNPs+FEP: 0.26, 0.37, 0.31 and 0.5 μg/mL	
					AgNPs+CAZ: 0.53, 0.5, 0.28 and 0.5 μg/mL	
Green synthesis of AgNPs from peel extract of *Chrysophyllum albidum* fruit	Almost spheric	28–90 nm	Tetracycline (TET) and ciprofloxacin (CIP)	Methicillin-resistant *S. aureus* (NCTC 12493), *E. coli* (ATCC 25922), *K. pneumoniae* (NCTC 13440), *B. subtilis* (ATCC 10004), *S. mutans* (ATCC 700610), *P. aeruginosa* (ATCC 4853) and *S. typhi* (ATCC 14028)	Alb-AgNPs with TET revealed synergistic effect against *K. pneumoniae, S. mutans* and *B. subtilis*, with partial synergy observed against MRSA and *P. aeruginosa*, antagonistic effect against *E. coli* and *S. typhi.* Alb-AgNPs with CIP revealed synergistic effect against MRSA and *P. aeruginosa,* partial synergy for *K. pneumoniae*, antagonistic effect against *E. coli* and *S. typhi,* and additive and indifferent effect for *B. subtilis* and *S. mutans* Alb-AgNPs: 125, 15.62, 15.62, 1,000, 250, 15.62 and 1,000 μg/mL	Ankudze and Neglo (2023)
Green synthesis of AgNPs using aqueous extract of *Salvia officinalis* leaves	Spheric	5–60 nm	Colistine	*A. baumannii* (ATCC 43498), *K. pneumoniae* (ATCC 700603), *E. coli* (ATCC 25922), *E. cloacae* (ATCC 13047), *P. aeruginosa* (ATCC 9027) and *S. typhimurium* (ATCC 14023)	AgNPs showed synergism when combined with colistin against *E. cloacae, E. coli, K. pneumoniae* and *S. typhimurium* in 53.63, 35.76, 35.19 and 33.06%, respectively. However, weak synergy was observed against the *P. aeruginosa* strain at 13.75%	Yassin et al. (2022)
Green synthesis of Ag/AgCl nanoparticles derived from *Chara* algae (*C. vulgaris*) extract	Spheric	AgNPs 6.4 nm and AgCl NPs 29.72 nm	Cloxacillin (CX), vancomycin (VA), gentamicin (CN), ciprofloxacin (CIP), tobramycin (TOB), erythromycin (E) and cefixime (CFM)	*S. aureus, E. coli, K. pneumoniae* and *P. aeruginosa*	Antibiotic efficacy improved by Ag/AgCl NPs. Zones of inhibition increased significantly against *S. aureus* with cloxacillin (CX) and cefixime (CFM) NPs. Additive effects for NPs with CFM. Against *E. coli*, the antibacterial action of the isolated antibiotics was limited, but the combination with Ag/AgCl NPs resulted in significant inhibition. For *K. pneumoniae*, the combination of erythromycin (E) with NPs was partially synergistic and showed inhibition. For *P. aeruginosa*, the antibacterial action was significantly increased with Ag/AgCl NPs, with partial synergy for a combination with vancomycin (VA) and an additive effect for cloxacillin (CX), erythromycin (E) and cefixime (CFM)	Hassan et al. (2021)
Green AgNPs were synthesized by *Ligustrum lucidum* leaf extract	Almost spheric	13 nm	Epoxiconazole	*S. turcica*	The prominent synergistic antifungal effect occurred at 8:2 and 9:1 for AgNPs and epoxiconazole, and the inhibition toxicity ratio reached 1.22 and 1.24, respectively St-AgNPs: 170.20 μg/mL	Huang et al. (2020)
Tween-stabilized AgNPs	Not specified	20–40 nm	Gentamicin	Clinical strains of Gentamicin-resistant *S. epidermidis*	The combination of AgNPs and gentamicin allowed the MIC to be reduced by 16 times SNPs + Gentamicin: 87-350 μg/mL	Mazur et al. (2020)
Green synthesis of AgNPs manufactured from aqueous extracts of *Anastatica hierochuntica* L. (Kaff Maryam; An-AgNPs) and *Artemisia absinthium* seeds (Ar-AgNPs)	Spheric and semi-spherical	*An*-AgNPs 114 and *Ar*-AgNPs125.5	Bacitracin (B), ciprofloxacin (CIP), tetracycline (TE), cefixime (CFM), fluconazole (FL) and metronidazole (ME)	*P. aeruginosa, E. coli* and S*. aureus*	Ar-AgNPs have significantly improved the efficacy of antibiotics, especially against *P. aeruginosa*. There were notable synergistic effects with cefixime, ciprofloxacin, and bacitracin. Ar-AgNPs showed activity against fungi when combined with fluconazole or metronidazole, and greater efficacy was observed against *S. aureus*. The MIC values of Ar-AgNPs ranged from 25 to 50% against all microbes tested, being most effective against *S. aureus.* An-AgNPs surpassed the isolated activity of antibiotics against *E. coli* and *S. aureus*. In *P. aeruginosa*, An-AgNPs were effective except with bacitracin. For *C. albicans*, An-AgNPs were less effective than fluconazole and metronidazole. Combinations of An-AgNPs with some antibiotics have shown synergistic effects against *E. coli* and *S. aureus*. In *P. aeruginosa*, there was synergy with ciprofloxacin, but not with tetracycline or cefixime. The combination of bacitracin and AgNPs had a significant synergistic effect. For *C. albicans*, combinations with fluconazole or metronidazole reduced the effectiveness of these antifungals. Some antibiotic-AgNP combinations suggest antagonistic effects	Aabed and Mohammed (2021)
					*An*-AgNPs: 50, 50 and 50%	
					*Ar*-AgNPs: 50, 50, and 25%	
AgNPs synthesized with *Artemisia argyi* leaf extract	Not specified	77.6 nm	Domiphen	*A. baumannii* (ATCC 19606), *S. aureus* (ATCC 6538), *E. coli* (8099) and *C. albicans* (ATCC 10231)	The combination of AgNPs synthesized with *Artemisia argyi* leaf extract and domiphen has synergistic anti-biofilm effects and could reduce the dosage of each antimicrobial drug	Hu et al. (2021)
					MICs AgNPs/Domiphen: 2/2, 2/4, 2/8 and 4/4 μg/ML	
					FICs AgNPs+Domiphen: 0.5, 0.375, 0.1875 and 0.3125	
Biosynthesis of AgNPs using Eurotium cristatum, isolated from Fuzhuan tea-brick	Spheric	15–20 nm	Vancomycin, oleandomycin, ceftazidime, rifampicin, penicillin G, neomycin, cephazolin, novobiocin, carbenicillin, lincomycin, tetracycline and erythromycin	*P. aeruginosa, C. albicans, S. aureus, E. coli* and *B. subtilis*	AgNPs combined with antibiotics produced an inhibition on pathogenic strains greater than the sum of their individual effects. There was a 2.5-fold greater zone of inhibition against *C. albicans* when used together	Lin et al. (2020)
Biosynthesis of AgNPs using *Amaranthus retroflexus* leaf extract broth as a reducing and stabilizing agent	Spheric	48 nm	Ciprofloxacin	*E. coli, P. aeruginosa, P. syringae* and *X. oryzae*	The combination of AgNPs with Ciprofloxacin reduced the MIC of the antibiotic from 0.125 μg/mL to 0.0625 μg/mL for *P. aeruginosa* and that against *P. syringae* decreased from 0.25 to 0.0625 μg/mL in combination with 6, 25, 12.5, and 25 μg/mL of AgNPs	Nikparast and Saliani (2018)
AgNPs synthesized from cell-free supernatant of *Klebsiella pneumoniae*	Almost spheric	20 nm	Gentamicin and chloramphenicol	*E. faecalis*	There was a noticeable increase in the antibacterial activities of chloramphenicol and gentamicin when measuring the diameter of the inhibition zone of the antibiotics combined with the nanoparticles	Katva et al. (2017)
Vancomycin-loaded AgNPs stabilized by trisodium citrate	Spheric	25 nm	Vancomycin	*S. aureus* and *E. coli*	The zone of inhibition for *S. aureus* was increased from 16 to 26.5 mm, while for *E. coli* from 0 to 7.5 mm. After treated with Vancomycin-AgNPs, vancomycin-resistant *E. coli* showed sensitivity to the AgNPs-drug conjugate. Furthermore, vancomycin-sensitive *S. aureus* became even more sensitive. In this way, the synergistic effect was highlighted	Kaur et al. (2019)
AgNPs synthesized by *Fusarium oxysporum* (AgNP bio) in combination with simvastatin	Almost spheric	77.68 ± 33.95 nm	Simvastatin	Methicillin-sensitive *S. aureus* (MRSA; ATCC 25923) and (ATCC 29213), MRSA (N315), MRSA BEC 9393, *E. coli* (ATCC 25922), and *E. coli* (ESBL 176) extended-spectrum beta-lactamases.	A synergistic effect of simvastatin-AgNP on antibacterial activity against MRSA strains was demonstrated, in addition to showing antibacterial activity against ESBL-producing *E. coli*	Figueiredo et al. (2019)
					AgNPbio+Simvastatin: 0.212, 0.212, 0.212, 0,212, 0.106 and 0.106 mg/mL	

*Acinetobacter baumannii, Bacillus subtilis, Candida albicans, Enterococcus faecalis, Escherichia coli, Klebsiella pneumoniae, Proteus vulgaris, Pseudomonas aeruginosa, Pseudomonas syringae, Salmonella typhi, Setosphaeria turcica, Staphylococcus aureus, Streptococcus mutans, Staphylococcus epidermidis, Vibrio cholerae*, and *Xanthomonas oryzae*.

In summary, the diverse applications of AgNPs as antimicrobial agents encompass a wide range of microorganisms of significant interest in hospital setting, particularly for hospital-acquired infections. By exploring these applications, it is possible to advance the search for innovative solutions to combat and reduce microbial infections and MDR strains through the use of nanotechnology and the unique properties of silver. Current applications of nanomedicine demonstrate significant advancements in the field. These technologies improve existing medical products, offering substantial benefits for patients and healthcare professionals in terms of efficacy, safety and infection control.

## 4 Silver nanoparticles applied as antibiofilm agents

**Figure 1:**
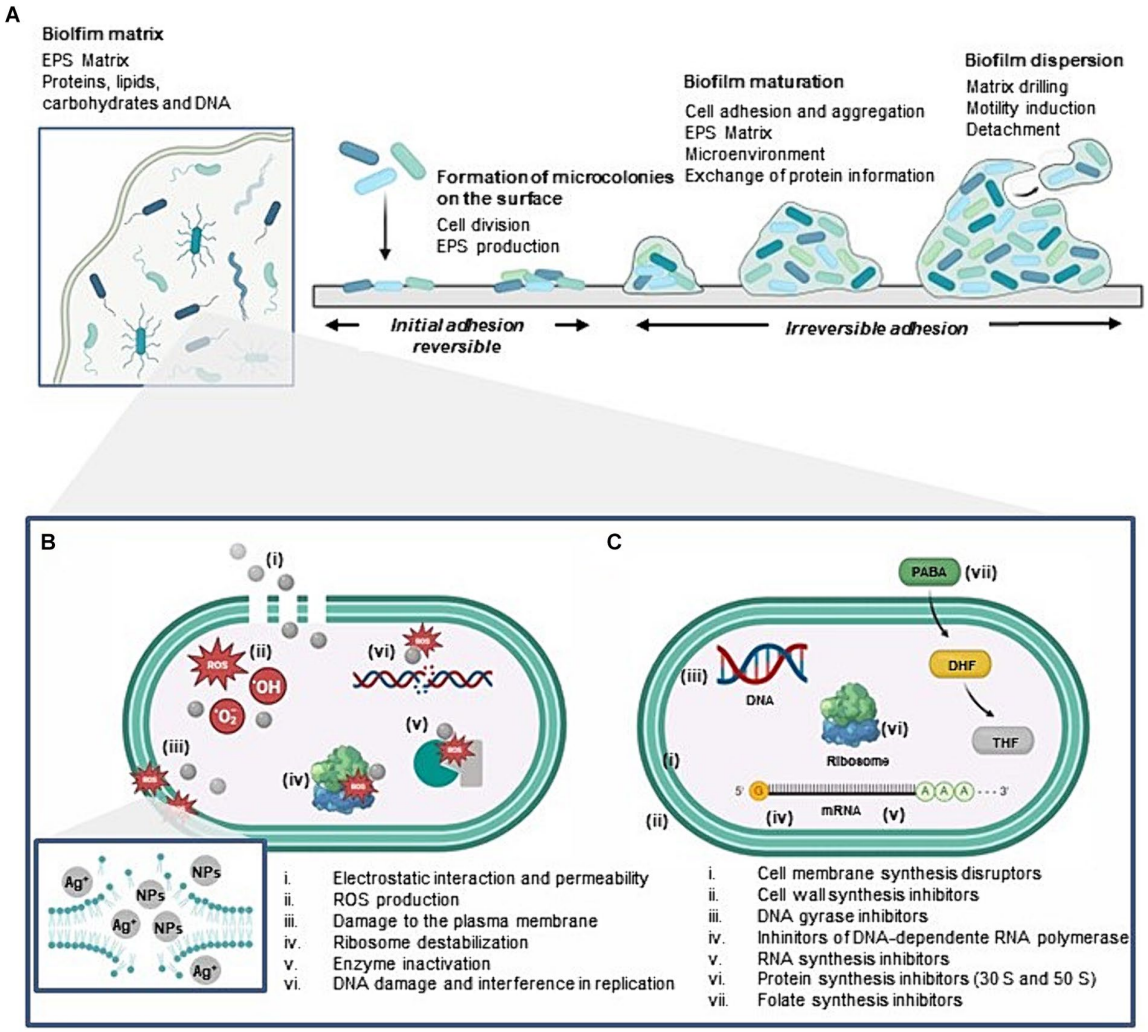
Graphical representation of bacterial biofilm formation **(A)** and comparison between the mechanism of action of AgNPs **(B)** and classes of antibiotics in standard use **(C)** in bacteria: Scattered colonies are deposited on the surface where the biofilm will begin to form. Bacteria adhere to the surface in a reversible way mediated by physicochemical interactions. Bacterial multiplication and formation of microcolonies follow as a consequence of cell division and production of extracellular polymeric substances (EPS). These substances forms an extracellular matrix that surrounds and protects the bacterial cells, providing adherence to the biofilm. As the biofilm matures, bacteria accumulate and more EPS are produced, increasing the biofilm. Eventually, parts of the biofilm may detach from the surface and disperse, carrying bacterial colonies that can colonize new surfaces and begin the biofilm formation process again.

Bacteria can be found in a mobile, also called planktonic state, being free to disperse and go in search of the nutrients present in their microenvironment or attached to surfaces in a community of microorganisms, called biofilm. In biofilms, bacteria can be protected against harmful conditions, as antibiotic agents, by an extracellular polymeric matrix composed of polysaccharides, protein, lipid and extracellular DNA, produced to promote their adhesion. This makes it difficult for antimicrobial substances to penetrate through the biofilms, making these multicellular structures an additional challenge to overcome microbial contamination (Figure 1). Surgical site infections are common examples of biofilms infections occasioned by surgery procedures that can increase the recovery time, medical costs or even result in death (Bajire et al., 2023; Al-Sawarees et al., 2024).

Besides the increased defense obtained with this polymeric matrix, that can act as a semipermeable barrier, some phenotype properties of these microorganisms are affected by the formation of these structures, such as the growth rate and gene transcription, which are commonly increased, allowing the formation of biofilms within a few hours. Furthermore, the bacterial genetic diversity inside the biofilm is mediated by the communication between the cells close to the surface (in contact to the outer environment) and the inner cells through the exchange of plasmids, which strongly contributes to the development of antimicrobial resistance (Pon Janani et al., 2022; Chauhan et al., 2023).

Although biofilms are statically attached to inert or living surfaces, when there is a reduction in the nutrients supply or other changes in physical or chemical factors, the biofilms can propagate by the detachment of clumps of cells or individual organisms, in a process known as seeding dispersal, allowing the proliferation of the infection. Due to all these differences between planktonic and static multicellular structures, biofilm infections may require different agents or approaches for treatment, hence the need for the development of antibiofilm agents (Halsted et al., 2022; Pon Janani et al., 2022; Bajire et al., 2023).

Among the established options to control biofilms formation, we can include ultraviolet irradiation, chlorine and chloramine treatment, hydrogen peroxide and nitrous oxide. However, in some situations these agents cannot be used because of their toxicity, other potential damages or even due to the antibiotic resistance. The environment of biofilms presents a gradient of oxygen content, decreasing from the surface to its deep layers due to the oxygen consumption by the first bacteria, forming anaerobic niches. Additionally, differences in pH between the different regions of these structures can be noticed, resulting in a variety of cells that may differ genotypically and phenotypically. Since higher levels of conventional antimicrobials are required to combat biofilm infections, the risk of systemic toxicity also rises. Nanoscale antimicrobial agents emerge as a promising solution, aiming to enhance antibiofilm activity without inducing harmful side effects (Al-Sawarees et al., 2024.; Chauhan et al., 2023).

When applying metallic nanomaterials to control biofilms growth, three critical steps are known to govern their action; (i) the interactions with the biofilm surface, affected by NPs charge; (ii) entry in the biofilm, dependent on nanomaterials properties such as surface chemistry and charge, size and concentration, as well as to biofilm surface composition and maturity; and (iii) the ability of NPs and their ions to migrate internally to interact with all the components of the biofilm (Chauhan et al., 2023).

AgNPs have been extensively studied as antibiofilm agents, even being used commercially as a coating material to prevent the infection of surfaces of medical supplies as catheters and wound dressings (Al-Sawarees et al., 2024). Table 3 presents the results of some applications of AgNPs as antibiofilm agents.

**Table 3:** Silver nanoparticle formulations applied in reducing and inhibiting the growth of bacterial biofilms.

Synthesis method	Other components	Biofilm microorganisms	Surface/sample treated	Average size (nm)	Charge (mV)	Results	References
Chemical reduction	Triclosan	*S. aureus* (ATCC25913)	Surgical Sutures (PDS® II and PDS Plus® Ethicon)	24.3 ± 6.2	−34.6 ± 4.7	Inhibited *S. aureus* and *E. coli* biofilms, with synergistic effect between AgNPs and TCA (measured by Checkerboard Microdilution Assay), enhancing the surgical suture performance	Al-Sawarees et al. (2024)
	Trans-cinnamaldehyde (TCA)	*E. coli* (ATCC8739)					
Electrical arc discharge	Carbon shell	*-*	Plates	17	−25.0 (pH 7)	Although the NPs were not tested against biofilms, it was noticed the inhibition of mrkA (biofilm forming gene) genetic expression in planktonic *P. aeruginosa*.	Elwakil et al. (2024)
					−43.8 (pH 10)		
Biosynthesis [supernatant of *B. licheniformis (BL)*, *B. cereus (BC)* and *F. oxysporum (FO)*]	-	*P. aeruginosa* (PTCC 1599 and Pa3)	96-well round-bottom microplate	19.41–27.03 (BL)	-	Reduced biofilm formation, changed the relative expression of biofilm-associated genes in a concentration dependent manner	Esfahani et al. (2023)
				28.49–43.62 (BC)			
				59.99–79.14 (FO)			
Biosynthesis [extracts of *Cystoseira baccata* (CB) and *Cystoseira tamariscifolia* (CT)]	-	*S. aureus* (ATCC 23235)	96-well plates	21.7 ± 6.2 (Ag@CB)	−34.3 ± 1.0 (Ag@CB)	AgNPs presented a MIC similar or lower to that of silver ions and lower to ampicillin and kanamycin (reference antibiotics)	Fernandes et al. (2023)
		*P. aeruginosa* (PAO1)		22.0 ± 1.4 (Ag@CT)	−26.9 ± 0.7 (Ag@CT)		
Chemical reduction	Mercaptopoly (ethylene glycol) carboxylic (mPEG)	*P. aeruginosa* (Pa1016)	Silicone disks	18.9 ± 2.2 (Ag)	−46.3 ± 6.3 (Ag)	Combination of AgNPs and hyperthermia significantly reduced biofilm formation	Palau et al. (2023)
	Amikacin (AK)			33.3 ± 8.2 (Ag/mPEG)	−30.5 ± 5.6 (Ag/mPEG)		
				39.8 ± 10.3 (Ag/mPEG/AK)	−21.7 ± 3.7 (Ag/mPEG/AK)		
Biosynthesis (supernatant of *P. aeruginosa* PA75)	Colistin	*K. pneumoniae*	18 mm cell slides	13.9 ± 4.0	−31.0 ± 8.1	Presented biofilm inhibition activity and mature biofilm bactericidal activity	Xia et al. (2023)
Phytosynthesis (extract of *Corymbia maculata* leaves)	Gentamycin, ciprofloxacin, imipenem, aztreonam, ceftazidime, linezolid, mupirocin, teicoplanin, vancomycin, cefoxitin, azithromycin, doxycycline, clindamycin, sulfamethoxazole-trimethoprim and augmentin	Clinical isolates of *P. aeruginosa*, *S. aureus* and methicillin-resistant *S. aureus* (MRSA) isolated from chronic rhinosinusitis patients	96-well microplates	40	-	Several additive effects were obtained, while synergistic antibacterial activity was observed when combining AgNPs and gentamicin against *P. aeruginosa* and MRSA biofilms	Feizi et al. (2022)
Chemical co-precipitation	Thiosemicarbazide (TSC) and glutamic acid (linker)	*P. aeruginosa* isolates	96-well plate	40–60	−13 (AgNPs)	AgNPs-TSC decreased up to 60% the expression of the pslA (polysaccharide synthesis locus) gene, reducing biofilm formation.	Honarmand et al. (2022)
					32.6 (AgNPs-TSC)		
Phytosynthesis (aqueous extract of *Koelreuteria paniculate* leaves)	-	*P. aeruginosa* PAO1	96-well polystyrene microtiter plate	30 ± 5	~−12	AgNPs effectively inhibited the formation of biofilm of PAO1, the production of several virulence factors and the expression of quorum signaling-regulated genes.	Kumar et al. (2022)
Phytosynthesis (grapevine canes extract from viticulture waste)	Grapevine canes extract	*C. albicans* (ATCC 10231)	96-well polystyrene microtiter plates	34.4 ± 1.4 to 103.4 ± 4.9	−30.0 ± 2.4 to −21.2 ± 5.4	Although all AgNPs dispersions were active against biofilms, larger and polydisperse NPs were more effective and a synergistic action of nanoparticles with biologically active extract compounds was noticed	Miškovská et al. (2022)
Phytosynthesis (*Aloe vera* extract)	-	*E. coli* (U12)	Microliter plate wells and urinary catheters	9.26–31.18	-	Completely inhibited *E. coli* biofilm formation and gradually reduced the production of biofilm with increasing the concentrations of AgNPs	Selem et al. (2022)
Chemical reduction	Chlorin e6-modified polyethyleneimine (PEI-Ce6)	*S. aureus* (ATCC 29213)	96-well microculture plate	10 (AgNPs)	−40 (AgNPs)	AgNPs and photodynamic therapy exhibited a synergistic antibacterial effect with the almost complete eradication of *E. coli* biofilm	Sun et al. (2022)
		*E. coli* (ATCC 25922)		60 (AgNPs/PEI-Ce6)	−20 to 35 (AgNPs/PEI-Ce6)		
Phytosynthesis (*Cinnamon cassia* barks and *Zingiber ofcinale* root extracts)	-	*E. faecalis* (89%) and *E. faecium* (11%) from biofilm-formed enterococcal urinary tract clinical isolates	Foley urinary catheter	55.7 ± 0.9 (Ag)	−9.35 (Ag)	Antibioflm activity at sub-MIC values (1/2, 1/4, 1/8 MIC) was concentration dependent and ginger AgNPs had the most potent antibacterial and antiadherent activities	Swidan et al. (2022)
				8.7 ± 0.7 (Ag/cinnamon)	−38.7 (Ag/cinnamon)		
				42.0 ± 7.2 (Ag/ginger)	−28.4 (Ag/ginger)		
-	-	*S. epidermidis* (ATCC 12228, ATCC 35983, and ATCC 35984)	96-well microculture plate	10	-	Biofilm formation reduced for all strains, with *icaADBC* operon and *icaR* genes expression reduction.	Swolana et al. (2022)
Phytosynthesis (*Bothriochloa laguroides* aqueous extract)	-	*Y. enterocolitica* (8081 and ME110)	96 round-bottomed welled polystyrene microtiter plates	8	-	Inhibited almost completely biofilms formation and eradicated more than 80% of mature biofilms at [AgNPs] > MIC	Toranzo et al. (2022)
		*S. aureus* (43300 and 29213)					
Laser Ablation in Liquid	Magnetic elements (Co and Fe)	*E. cloacae* (CCM 1903)	24-well plates	2.9–6.4 (Ag)	−32.9 ± 2.8 (Ag)	AgNPs can perforate biofilms through magnetophoretic migration by the application of a magnetic field and increase the membrane lipid peroxidation biomarkers through the formation of ROS	Torres-Mendieta et al. (2022)
		*P. putida* (CCM 7156)					
		*E. faecalis* (CCM 4224)		2.9–8.0 (AgCo)	−41.5 ± 0.3 (AgCo)		
		*B. subtilis* (CCM 1999)		2.9–13.5 (AgFe)	−38.6 ± 0.5 (AgFe)		
Phytosynthesis (*Zataria multiflora*)	-	*S. aureus* (ATCC 25923)	96-well microtiter plate	48.5 (pH 6)	-	Plant-based AgNPs showed great biofilm inhibition for all the tested concentrations, with a better performance than commercial AgNPs, aqueous silver nitrate solution and gentamicin	Barabadi et al. (2021)
				56.0 (pH 7)			
				22.5 (pH8)			
				25.5 (pH 9)			
Chemical reduction	-	Oral biofilms from subjects with or without active dental caries (mainly *S. mutans and S. sobrinus*)	96-well plates	5.2 ± 1.2	−48.4 ± 6.9	Both AgNPs had significant antimicrobial effects against all samples of dental plaque, with better antibiofilm activity for the smaller samples and against biofilms from patients without caries. However, 2% chlorhexidine results were better than AgNPs	Jiménez-Ramírez et al. (2021)
				37.4 ± 3.6	−52.6 ± 8.5		
Chemical reduction (Commercial samples from NanoComposix)	-	Isolates of *S. pseudintermedius* obtained from dogs with otitis externa	Flat-bottom, clear plastic cell culture plates with lids	10	-	AgNPs displayed dose-dependent antibiofilm activity and reduced biofilm formation at concentrations of 20 and 10 μg/mL, with less bacterial slime formation when treated with the 20 μg/mL AgNPs suspension	(Seo et al., 2021)
Biosynthesis (cell-free supernatant from putative *Cedecea* sp. strain isolated from soil)	Antimicrobial peptides	*E. coli* (UTI 89)	15 mm cover glass	10–40	−15.3	AgNPs presented better results against gram-negative bacteria biofilms and showed to be stable for periods exceeding 1 year	Singh et al. (2021)
		*P. aeruginosa* (PAO1)					
		*S. epidermidis* (ATCC 35984)					
		*S. aureus* (CCUG 10778)					
Chemical reduction (sodium citrate)	Antimicrobial peptides and polydopamine (AMP@PDA)	*S. aureus* ATCC 25923*, E. coli* ATCC 25922 and *P. aeruginosa*	96-well plates	150–200	-	Modification of AgNPs with AMP@PDA improved biocompatibility and antimicrobial activity of the nanocomposites, with antibiofilm action against all tested strains and decrease of mRNA expression of biofilm-related genes.	Xu et al. (2021)

*Candida albicans, Enterococcus faecalis, Enterococcus faecium, Klebsiella pneumoniae, Pseudomonas aeruginosa, Pseudomonas putida, Staphylococcus epidermidis, Staphylococcus pseudointermedius, Streptococcus mutans, Streptococcus sobrinus*, and *Yersinia enterocolitica.*

Table 3 presents a variety of synthesis methods applied in the production of AgNPs with antibiofilm properties. Traditional chemical reduction procedures typically involve hazardous and sometimes expensive substances, necessitating purification steps to remove excess unreacted reagents and reduce toxicity. This increases both the time and cost of nanoparticle production. In contrast, green synthetic methods not only make the produced nanoparticles safer and more cost-effective but also environmentally friendly and conducive to scalability. These methods utilize natural compounds such as proteins, reducing carbohydrates, polyphenols, flavonoids, terpenes, and other secondary metabolites to reduce metal ions and/or act as capping agents. Two prominent classes of green synthesis for producing antibiofilm AgNPs have emerged: biosynthesis and phytosynthesis. Biosynthesis involves algae, microorganisms, or their cell-free extracts (filtrates or supernatants), while phytosynthesis utilizes plant extracts (Miškovská et al., 2022; Selem et al., 2022; Swidan et al., 2022; Toranzo et al., 2022; Esfahani et al., 2023; Fernandes et al., 2023; Xia et al., 2023).

Aiming to develop a sustainable synthesis method for AgNPs, Miškovská et al. (2022) applied a hydroethanolic solution of grape canes, a waste product of vine growing, to sustainably produce mono or polydisperse NPs suspensions that were effective against biofilms, also revealing a synergy between the AgNPs and the plant extract.

Fernandes et al. (2023) used microalgae, brown seaweeds, to biosynthesize AgNPs that presented better action against biofilms than the reference antibiotics kanamycin and ampicillin, while Esfahani et al. (2023) and Xia et al. (2023) applied biological compounds (bacterial or fungal metabolites) from the supernatant or biomass of microorganisms as reducing, stabilizer, and dispersant agents, both obtaining AgNPs with good results in biofilm control.

Physical methods can also be applied in AgNPs production. This class of synthesis methods can produce NPs with minimal impurities and, sometimes, in a most effective way than chemical and biological methods. One physical method applied for antibiofilm AgNPs production is the laser ablation in liquid, a low-cost and eco-friendly procedure in which a metal foil (or more than one, when producing multi element metallic NPs) is immersed in a solvent, followed by ablation with a laser beam, which results in NPs free of organic contaminants or capping agents, needing only of purification steps to change the solvent, for example, from a hydroethanolic solution to water (Torres-Mendieta et al., 2022).

Electrical arc discharge (EAD) is another example of this class in which two electrodes are positioned inside an open vessel containing ultra-pure water and, with the initiation of the arc discharge the silver electrode vaporizes being then quenched by the cold solution, forming nanoparticles (Tseng et al., 2011; Elwakil et al., 2024).

Although Elwakil et al. (2024) assessed the antimicrobial activity of the AgNPs covered with carbon shells produced by EAD only against planktonic bacteria, the authors reported the inhibition of mrkA (biofilm forming gene), fimH (virulence adhesion gene) and rmpA (mucoid factor encoding gene) genetic expression in *P. aeruginosa*, affecting its intracellular signaling pathway. Honarmand et al. (2022), in turn, observed the importance of inhibiting the polysaccharide synthesis locus (psl) gene for *P. aeruginosa* biofilm formation control. The authors produced AgNPs and conjugated it with TSC, antimicrobial compound that did not present an expressive effect in gene inhibition, but enhanced the antibiofilm activity when conjugated with the NPs. Since Psl, along with Pel polysaccharide are responsible for the formation of the primary matrix of biofilm, this effect of partial inhibition can be understood.

In view to interfere in quorum sensing (QS)-regulated *P. aeruginosa* biofilm formation, AgNPs were phytochemically prepared using aqueous extract of *K. paniculata* leaves, an anti-QS substance. The NPs reduced biofilm formation of a multidrug-resistant model strain of *P. aeruginosa* almost completely, downregulating several QS-virulence factors, such as pyocyanin, pyochelin, alginate, protease and rhamnolipid, and QS-regulated genes (lasR, rhlR, rhlI, lasI, lasA, lasB, rhlA, and rhlB; Kumar et al., 2022).

Xu et al. (2021) produced nanocomposites of AgNPs modified with antimicrobial peptides and polydopamine that were able to improve both HEK293T cells viability and antibacterial activity against *S. aureus, E. coli*, and *P. aeruginosa*. Thickness, biomass and the semi-quantitative analysis of crystal violet staining results indicated an effective reduction of biofilms of all strains, with the reduction of the mRNA expression of biofilm-related genes las I, rh II and fim H.

The size of AgNPs significantly impacts their ability to combat biofilms. The dense extracellular matrix of biofilms acts as a barrier to NPs penetration. Moreover, smaller AgNPs possess a greater surface area, allowing for increased interaction with biofilms and potentially enhanced antibiofilm activity (Selem et al., 2022). As shown in Table 3, most antibiofilm AgNPs are under 100 nm, with many falling below 50 nm. Polydispersity also plays a role, as Miškovská et al. (2022) noted that polydisperse NPs were more effective than monodisperse suspensions of AgNPs in controlling *C. albicans* biofilms.

Fernandes et al. (2023) compared the effectiveness of silver nitrate and AgNPs against *S. aureus* and *P. aeruginosa* biofilms, finding similar or lower MIC values for the phytosynthesized NPs. This suggests that silver ions, released from AgNPs play a crucial role in inhibiting microbial growth within biofilms, overcoming the compact biofilm structure that impedes antibiotic penetration.

Many microorganisms developed resistant strains that are not susceptible to the classic antibiotics. In these cases, a common strategy is the combination of antimicrobial agents aiming to obtain a synergistic effect, since these components can present complementary mechanisms to inhibit microbial growth and form stable complexes via weak intermolecular interactions that prolong their release and reduce their potential local toxicity (Feizi et al., 2022; Miškovská et al., 2022; Torres-Mendieta et al., 2022; Palau et al., 2023; Xia et al., 2023; Al-Sawarees et al., 2024). This approach can include mixing AgNPs with classical antibiotics (Feizi et al., 2022; Palau et al., 2023; Xia et al., 2023), plants extract rich in bioactive molecules as antioxidants (Miškovská et al., 2022) or magnetic elements (Torres-Mendieta et al., 2022), which allows the use of magnetic fields to damage biofilms, promoting NPs penetration by highways created via mechanical disruption without additional associated risks.

Al-Sawarees et al. (2024) mixed AgNPs with Triclosan (5-chloro-2-(2,4-dichlorophenoxy) phenol), a synthetic antimicrobial compound used in a variety of consumer products, including hygiene products and to coat medical supplies as sutures (PDS Plus®, Vicryl Plus® and Monocryl 250 Plus®) or trans-cinnamaldehyde (TCA), a phytochemical extracted from cinnamon with antimicrobial activity and generally recognized as safe by U.S. Food and Drug Administration (FDA). For both combinations the AgNPs effect against *S. aureus* and *E. coli* biofilms formed in surgical sutures was improved, with additive effect when combining the NPs to Triclosan and synergistic effect when used with TCA.

Besides combining substances, an approach applied in biofilms control involves the use of hyperthermia, i.e., the use of heat as a therapeutic method to control microbial growth. Hyperthermia is specially used in cancer treatment and involves cell death by local heating (41°C to 50°C). Palau et al. (2023) observed important biofilm reduction combining AgNPs functionalized with mercaptopoly (ethylene glycol) carboxylic and acid amikacin to hyperthermia, significantly reducing biofilm formation by *P. aeruginosa*.

Another well-established approach for microbial control is the photodynamic therapy (PDT). In this case, a photosensitizer agent is used to produce ROS or singlet oxygen when activated by light irradiation (typically UV–vis or near infrared light) in the presence of oxygen, since these species eradicate microorganisms via oxidative damage of cell wall or membranes, cytoplasm leakage, lipid peroxidation, metabolic inhibition and also damages to mitochondria, membrane transport system or DNA (Pon Janani et al., 2022). Sun et al. (2022) coated negatively charged AgNPs (−40 mV) with positively charged polyethylenimine (PEI) and chlorin e6 (Ce6), a natural molecule approved by FDA and commonly used as a photosensitizer. Although AgNPs and PEI-Ce6, in separate, were able to reduce the microbial adhesion, when combined, this system completely inhibited the biofilm of *E. coli* formation and significantly reduced *S. aureus* biofilm formation. The exposure of biofilms of *S. aureus* and *E. coli* to AgNPs-PEI-Ce6 suspensions in combination to PDT significantly reduced the microbial survival *in vitro*, and in mice both PEI-Ce6 and AgNPs-PEI-Ce6 promoted a faster recovery of *S. aureus* wound infection, showing the benefits of combining NPs and PDT.

To mimic real-world infections more accurately, researchers (Jiménez-Ramírez et al., 2021; Seo et al., 2021; Feizi et al., 2022; Swidan et al., 2022), utilized biofilms derived from clinical isolates. These biofilms often harbor multiple bacterial strains, requiring antibiofilm agents with diverse modes of action. Similarly, to treating standard reference strains, studies have shown that AgNPs are effective against biofilms formed by clinical isolates, demonstrating similar success to those observed with standard lab strains. For instance, Jiménez-Ramírez et al. (2021) investigated the impact of caries on oral biofilms (primarily composed of *S. mutans* and *S. sobrinus*). They found that AgNPs were more effective in controlling biofilms from individuals without caries.

The scenario of AgNPs in biofilm control shows that, despite the well-known properties of these NPs, the combination with other compounds or approaches can be a good option in the development of antibiofilm agents that efficiently control the formation or eliminate these bacteria structures, even when involving resistant strains.

## 5 Bacterial resistance to AgNPs and Ag ions

The fight against infections associated with the previously mentioned bacterial biofilms, particularly those caused by MDR strains has driven the development of novel approaches (Joshi et al., 2020). Persistent bacteria exhibit phenotypic changes that enable them to evade antimicrobial agents, often through genetic mutations (Salas-Orozco et al., 2022). Among the various bacterial species described in the literature, Gram-negative bacteria have shown the most significant resistance to silver. This resistance mechanism is partly due to presence of lipopolysaccharides (LPS) on their cell wall, which induce electrostatic repulsion with negatively charged AgNPs (Maillard and Hartemann, 2013). Factors contributing to silver resistance in bacteria involve chemical detoxification mechanisms and active efflux systems. These systems, such as P-type ATPases and cation/proton transporters facilitate the transport of silver ions into or out of bacterial cells. Additionally, silver ions can be reduced to their inactive elemental metallic form, decreasing their antimicrobial activity (Maillard and Hartemann, 2013).

Like the mechanism of action of AgNPs as an antimicrobial agent involves a series of factors and multiple ways of interacting, studies suggest that the mechanism of bacterial resistance to silver involves a combination of interrelated systems. Among the main mechanisms of bacterial resistance to silver described in the literature, the following may occur: induction of aggregation of silver nanoparticles; reduction of Ag^+^ ions to Ag^0^; contact protection and prevention of silver entry into cells; efflux of silver nanoparticles and Ag^+^ ions through cellular efflux pumps; activation of defense mechanisms and repair of cellular damage; biofilm defense; antibiotic-mediated silver resistance; mutation; modulating the surface charge of cell membranes and response to stress and vitality (Li and Xu, 2024).

Exposure to sublethal concentrations of AgNPs can trigger the development of resistance phenotypes due to oxidative stress and DNA damage. These phenotypes may arise through active and passive resistance mechanisms. Active resistance mechanisms involve the direct response of the bacterium to the antimicrobial stimulus, including the use of efflux pumps and genetic mutations. Passive resistance mechanisms involve intrinsic characteristics of the bacterium, such as the thickening of the cell wall and the formation of bacterial biofilm (Salas-Orozco et al., 2022).

Factors contributing to silver resistance in bacteria involve chemical detoxification mechanisms and active efflux systems. These systems, such as P-type ATPases and cation/proton transporters, play a role in transporting silver ions into or out of bacterial cells. Additionally, it is possible to reduce the silver concentration by converting the Ag^+^ ion to the inactive elemental metallic form Ag^0^, thus not presenting antimicrobial activity and other properties associated with the silver ion (Maillard and Hartemann, 2013).

AgNPs can interact with nucleic acids, aiding in the aggregation, formation, structure, and integrity of bacterial biofilm, as well as with membrane proteins and enzymes that promote homeostasis, as well as polysaccharides and lipids. These interactions can originate hydrophobic, Van der Waals, hydrogen bonding, π–π, ionic, or electrostatic, with the latter contributing most to the biocidal activity of nanoparticles (Joshi et al., 2020).

The ability of bacterial resistance to AgNPs was evidenced through TEM and UV–visible spectroscopy of nanoparticles. In the case of the *E. coli* CCM 3954 strain, classified as ‘silver-resistant’, aggregation and/or precipitation of AgNPs were induced. Flagellin, a structural protein of bacterial flagella responsible for bacterial locomotion, played a role in promoting the aggregation of AgNPs, resulting in the reduction of their antibacterial effect (Panáček et al., 2018).

A fact with few studies yet relates that resistance to antibiotics in bacteria can also lead to the development of resistance to silver. A study demonstrated that both AgNPs and Ag^+^ can remove mature *P. aeruginosa* biofilms depending on the concentration of these compounds, however, AgNPs and Ag^+^ at the same concentrations were not able to destroy the biofilm formed by gentamicin-resistant *P. aeruginosa* (Mann et al., 2021). Therefore, the use of inorganic antimicrobial agents and antibiotics needs to follow more rigorous protocols, as improper use can lead to the emergence of superbugs and compromise the action of various antibiotic therapy strategies (Yonathan et al., 2022).

Researchers studied the heredity and stability of resistance to AgNPs. By removing and reintroducing AgNPs in various growth passages, the hypermotile *E. coli* strain K-12 MG1655 maintained resistance, indicating a hereditary, irreversible, and permanent genetic alteration due to the expression of resistance genes, even in the absence of silver. Genomic analyses identified a missense mutation (R292L) in the *cusS* gene, which remained constant in all lineages. This mutation, located in the active site of dimerization and the histidine phosphotransfer domain, enhances silver ion efflux, thereby conferring resistance to AgNPs (Stabryla et al., 2021).

Analyzing the impact of bacterial motility on AgNPs resistance, researchers found that non-motile *E. coli* strain did not exhibit significant MIC increase upon repeated AgNPs exposition. Genomic analysis did not reveal the presence of resistance genes, indicating that resistance to AgNPs may be specific to strains with bacterial motility (Stabryla et al., 2021).

To assess physiological changes triggered by AgNPs exposure, *E. coli* pre-treated with AgNPs showed increased MIC and MBC values (of two to eight times) when subsequently exposed to antibiotics like aminoglycosides, penicillins, and phenicols compared to the group that did not receive pre-treatment with AgNPs, indicating adaptive responses mechanism that promove antimicrobial resistance (Kaweeteerawat et al., 2017).

Most studies determining the effectiveness of AgNPs as antimicrobials are performed in single-species cultures. However, bacteria are rarely found in a single-species group in the environment. Researchers have demonstrated mechanisms that allow the sharing of resistance between bacteria that are usually found in association. For example, multiple compounds secreted by *P. aeruginosa* have been found to increase the tolerance of *S. aureus* to silver, both microorganisms are found associated in severe chronic infections leading to increased virulence and tolerance to antibiotics. The secretion of *Pseudomonas* quinolone signal (PQS) or compounds directly controlled by PQS, as well as the amino acids serine and threonine, generate silver tolerance in *S. aureus*. These compounds probably affect the physiology and metabolism of *S. aureus* (PQS, serine and threonine) by decreasing the availability of silver in the medium through bonds (Monych Nadia and Turner Raymond, 2020).

Understanding these complex interactions between bacteria, AgNPs and silver ions is crucial for developing effective strategies against bacterial resistance. Such insights contribute to significant advancements in nanomedicine and infection control strategies.

## 6 Proteomic analysis of the antibacterial mechanism of action

Proteomics is a branch of molecular biology that studies the set of proteins expressed by an organism or cell in response to different conditions. For instance, when microorganisms are exposed to a certain antimicrobial agent such as AgNPs, proteomic analysis provides information about action and resistance mechanisms of these microorganisms to the antimicrobial agent.

Recent findings have revealed molecular processes underlying the action of AgNPs against microorganisms. Understanding these proteomic mechanisms can guide the development of new antimicrobial strategies. Here are some relevant considerations regarding the proteomics of microorganisms exposed to AgNPs which aid in understanding the results of such analyses.

### 6.1 Proteomic profile analysis

Proteomic analysis allows for the identification and quantification of specific proteins from a given organism or cell. Generally, each microorganism has a distinct identity (fingerprint) in its protein expression profile. When microorganisms are exposed to AgNPs, it is possible to investigate how these nanoparticles affect protein expression. Techniques such as two-dimensional gel electrophoresis (2D-PAGE) and mass spectrometry are frequently used to analyze the proteomic profile.

The study of microbial proteomics opens a universe of possibilities; different bacterial strains can exhibit different protein expression profiles even when exposed to a single formulation of silver nanoparticles. Similarly, a standard strain may display distinct proteomic profiles when exposed to different AgNPs. AgNPs are generally considered a unique type of antibacterial agent, but their physical and chemical properties determine the way they interact with bacterial cells, the mode of action, and the response of the exposed bacterial cell (Kędziora et al., 2021).

### 6.2 Mechanisms of action of AgNPs

AgNPs can interact with cellular proteins, leading to changes in protein expression. They can affect metabolic pathways, signaling processes, and stress responses. Proteomic analysis contributes to the identification of specific proteins involved in these mechanisms. Table 4 represents the data of proteins expressed in bacteria after treatment with AgNPs.

**Table 4:** Mechanism of action detected by proteomics: proteins expressed in bacteria after treatment with AgNPs.

Material	Size	AgNPs effect	Bacteria	Marker			Method	References
				Upregulation	Downregulation	Inhibition		
AgNPs reduced by branched cyclodextrin solution	5–20 nm	Inhibition of adhesion and motility, ROS, alteration of iron homeostasis, blockade of aerobic and anaerobic respiration, changes in quorum sensing (QS) and inhibition of the expression of virulent factors	*P. aeruginosa*	AntA, AntB and NarL	Dnr and NarX	ArcA, ArcD, NADH dehydrogenases (NuoE, NuoL and PA0949) and SdhC	TMT-labeled quantitative proteomic	Zhang et al. (2020)
AgNP stock solution	5–7 nm	Damage to bacteria, inhibition of peptidoglycan synthesis, damage to biofilm structure and bacterial adhesion, disturbances in QS, decreased bacterial proliferation and induction of ROS	*S. suis* (MDR)	S-ribosylhomocysteine lyase and cps2J	Chromosomal replication initiation protein DnaA, cell division initiation proteins FtsZ and DivIB and proteolytic subunit of the ATP-dependent Clp protease	Penicillin-binding proteins (PBPs)	iTRAQ-based proteomic analysis	Liu et al. (2023)
AgNPs reduced by sodium borohydride	10 nm	Inhibition of protein synthesis, disruption of antioxidant enzymes, induction of ROS and dysregulation of homeostasis of pentose phosphate oxidative pathway	*S. aureus*	-	-	Enzymes of the oxidative pentose phosphate pathway (oxPPP): 6-phosphogluconolactonase (Pgl) and 6-phosphogluconate dehydrogenase (6PGDH)	LC-GE-ICP-MS	Wang et al. (2021)
AgNPs reduced by DAMP (AgDAMP)	21.87 ± 0.06 nm	Alteration in the expression of regulators of protein synthesis, DNA replication, membrane transport, ROS and cell motility	*Salmonella*	ROS (A0A0K0HA36) and toxicity (A0A0K0HBQ0)	DEPs of protein biosynthesis (A0A724WMT2 and G5LUI4), efflux pump (A0A1S0Z705), phosphorylation transduction (A0A740TKQ2) and transcriptional modulation (A0A724WUQ6)	-	High-throughput sequencing	Wang et al. (2023)
Silver–lactoferrin (AgLTF) complex: ions Ag + and AgNPs	10–56 nm	Induction of ROS causing oxidative stress, decreased cellular respiration, increased bacterial stress and decreased energy resources	*E. faecalis, P. aeruginosa* and *S. aureus*	Diisopropyl sulfide, dimethyl trisulfide (resistance to bacterial stress), ethanol, acetone, 2-butanol (energy depletion), decanoic acid, nonane (continuous oxidative stress), fermentation pathways (oxygen depletion or transport chain compromise of electrons)	Acetyl-CoA, acetoacetate decarboxylation and butanediol fermentation, impaired butanoate and propanoate metabolism	-	MALDI-TOF-MS	Monedeiro-Milanowski et al. (2023)
AgNPs citrate-coated	45 nm	Induction of oxidative stress, effects causing alterations in structural proteins of ribosome and alterations in translation	*Pseudomonas* sp. M1	Superoxide dismutase (SOD), structural proteins of ribosome (30S S8, RpsH) and tRNA ligation proteins (30S S10, RpsJ)	Elongation factor proteins (Efp, Tsf and Tuf1)	-	SWATH-MS	Barros et al. (2019)
AgNPs	14 ± 0.3 nm	ROS, alterations in the expression of proteins in the formation and maturation of biofilm, bacterial growth, routes of utilization of glycose and reduction in thickness of the membrane and cell wall	*E. coli*	1-acylglycerol-3-phosphate O -acyltransferase, dissulfeto redutase, triosephosphate isomerase and UTP-glycose-1-phosphate uridylyltransferase and sensor kinase (RcsC)	MlaB, 2-methylcitrate synthase, guanosine-5′-triphosphate (GTP) cyclohydrolase II, phosphate acyltransferase, L, D-transpeptidase (LdtD), β-glucuronidase, phospholipase, malate dehydrogenase, YcgR and c-di-GMP	-	Label-free proteomic based on LC–MS/MS	Domingo et al. (2019)
AgNPs	-	Damage to cell membrane and induction of oxidative stress by ROS	*P. aeruginosa*	AtpE, PA2536, PA4504, OprH, OprD and OprC	PilP, PilX, FlgE, FliN, PA4133, Hmp, KatA, CcoP2, SodB, CcpA, RibC, EtfA and PiuC	Metal carriers, OprC (Cu), CcoO1 and CcoO2 (Fe), MgtE (Mg) and PA0372 (Zn)	iTRAQ and 2D-LC–MS/MS	Yan et al. (2018)

*Enterococcus faecalis, Escherichia coli, Pseudomonas aeruginosa, Staphylococcus aureus*, and *Streptococcus suis.*

In early reports of proteomic analyses, researchers identified a potential mode of action underlying the antibacterial action of AgNPs. An accumulation of protein precursors from the envelope was found in the proteomic signatures of *E. coli* cells treated with AgNPs, suggesting that AgNPs may interact with the bacterial membrane. Brief exposure of *E. coli* cells to AgNPs resulted in changes in the expression of envelope proteins (OmpA, OmpC, OmpF, OppA, MetQ) and heat shock proteins (IbpA, IbpB, and ribosomal subunit 30S S6). Additionally, AgNPs and Ag^+^ ions in the form of AgNO_3_ exhibited similar mechanisms of action targeting the membrane. AgNPs and Ag^+^ ions were used at nanomolar and micromolar levels, respectively, suggesting that AgNPs have significantly greater antimicrobial activity than Ag^+^ ions (Lok et al., 2006).

Studies conducted with *P. aeruginosa* (Gram-negative bacteria) treated with AgNPs yielded comprehensive proteomic responses, aiding in the elucidation of the antimicrobial mechanism. Proteomic analysis of bacteria treated with AgNPs revealed elevated levels of enzymes such as superoxide dismutase (SOD), catalase (CAT), and peroxidase (POD), as well as alkyl hydroperoxide reductase and hydroperoxide resistance proteins. There was also significant positive regulation of low-oxygen regulatory oxidases, such as subunits P2, N2, and O2 of cytochrome c oxidase type cbb3. This demonstrates that the primary mechanism of action of AgNPs involves the imbalance of oxidation and antioxidation processes and the failure to eliminate excess ROS (Liao et al., 2019). Another study identified 5 silver-binding proteins and 59 proteins regulated by silver (27 positively regulated proteins and 32 negatively regulated proteins). Proteomic profiling showed that the cell membrane was the primary target of AgNPs, leading to oxidative stress induced by ROS generation. The release of silver ions and specific particle effects synergistically contribute to the antibacterial action of AgNPs. Moreover, the same silver-binding proteins were obtained with AgNPs and silver ions, indicating that AgNPs likely affect the cell membrane and react with proteins, releasing silver ions. These results demonstrate that the antimicrobial activity of AgNPs is due to the synergistic action of dissolved silver ions release and specific particle effects (Yan et al., 2018).

In another investigation, proteomic analysis of AgNPs against multidrug-resistant *P. aeruginosa* analyzed antibiofilm mechanisms. Based on Tandem Mass Tag (TMT)-labeled quantitative proteomics, various antibiofilm mechanisms of AgNPs were revealed. These included inhibition of adhesion and motility, strong stimulation of oxidative stress response, disruption of iron homeostasis, blocking of energy production (both aerobic and anaerobic), and affecting quorum sensing detection. The latter is a communication mechanism used by bacteria to coordinate gene expression based on their population density. Proteomic analysis showed alterations in protein expression at different functional levels, suggesting that AgNPs may eliminate *P. aeruginosa* biofilms in multiple ways (Zhang et al., 2020).

Based on quantitative proteomic analysis (iTRAQ), it was discovered that AgNPs disrupt the natural morphology environment of *S. suis* and its biofilm. Several proteins related to peptidoglycan synthesis were negatively regulated, including penicillin-binding proteins (PBPs), glycosyltransferases, and LytR family transcriptional regulators, leading to bacterial membrane rupture. Additionally, AgNPs can prevent bacterial adhesion, interfere with QS system, and inhibit bacterial growth by targeting the cell division protein FtsZ and the chromosomal replication initiator protein DnaA. Increased oxidative stress is also a relevant factor contributing to bacterial death (Liu et al., 2023).

### 6.3 Applications

Conventional antibiotics have revolutionized the treatment of bacterial infections and led to the emergence of strains resistant to various antibiotics restricting antimicrobial molecules to hospital to avoid the development of superbugs that cannot be combated with the existing arsenal of antibiotics. The continuous and excessive use of such antibiotics has led to outbreaks of superbugs in hospitals and communities. In recent decades, silver has been used in medical treatments such as burns, wounds, and bacterial infections. Metallic silver, silver nitrate, and silver sulfadiazine have been used in these treatments. Nowadays, AgNPs and silver ions are used as antibacterial agents in the medical field in the form of nanoparticles and ions, with proven effectiveness against bacteria, fungi, and viruses. These nanostructures can still be modified or functionalized to be more effective than pure AgNPs. Size, shape, concentration, surface charge, and functionalization are the main properties that determine antimicrobial efficiency (Hamad et al., 2020). The broad-spectrum antimicrobial activity of nanomaterials compared to antibiotics may off a solution to combat MDR bacterial strains. For example, AgNPs act through various mechanisms, including overcoming antibiotic resistance mechanisms, damaging membranes, and preventing biofilm formation. When applied together with other antimicrobial molecules, therapy can be enhanced and the emergence of new MDR strains avoided (Alavi and Hamblin, 2023).

Spherical AgNPs showed efficacy against *Porphyromonas gingivalis* and *S. mutans* (MIC = 8 μg/mL) and significantly prevented the adhesion of *S. mutans in vitro*. Proteomic analysis showed that after a short period of treatment of bacteria with AgNPs, changes in the expression of various heat shock proteins and bacterial cell coating proteins were observed, suggesting that nanoparticles may penetrate the bacterial membrane leading to destruction. AgNPs can also cause a decrease in intracellular potassium, resulting in a reduction in ATP levels. Possible molecular targets of AgNPs may be thiol protein groups and the phospholipid part of the bacterial cell membrane. Therefore, AgNPs@*Abelmoschus esculentus* may be good candidates for oral hygiene agents to prevent periodontopathic conditions and dentures (Nie et al., 2023).

Researchers studied a strategy combining hollow mesoporous silica nano spheres (HMSN) loaded with AgNPs, vancomycin and hemin (HAVH) for the elimination of MDR bacteria in abscess therapy. Demonstrating that the multi-path antibacterial mechanisms of HAVH could reduce the risk of drug resistance development by addressing the limitation of conventional antibiotics. In addition to the strategy combining AgNPs with antibiotics (HAVH), NIR irradiation showed improved synergistic effects, suggesting the potential of combinatory strategies for treating MDR bacterial infection. Enrichment analysis of genetic ontology (GO) indicated that differentially expressed proteins were associated with biological processes, cellular components, and molecular functions, including changes in nucleotide binding sites, cytoplasm, and transcription processes (Yan et al., 2023).

### 6.4 Resistance and adaptation: a proteomic view/approach

Prolonged exposure to AgNPs can lead to the selection of resistant microorganisms. Proteomics can reveal how microorganisms adapt to AgNPs by modifying their protein expression. Thus, application studies should consider the possibility that AgNPs may increase antibiotic resistance by causing mutations in resistance genes (Alavi and Hamblin, 2023). A study using *E. faecalis* demonstrated the ability of these microorganisms to develop resistance to AgNPs. This resistance consists of the expression of multifaceted mechanisms. Proteomic analysis revealed that 1,080 proteins were unique to *E. faecalis* exposed to AgNPs compared to 8 identified in the control sample. Among the affected biological processes and molecular functions are oxidative stress (superoxide dismutase and peroxiredoxin AhpC); transcriptional factors (Cro, Lux, and Rex); universal stress protein; and fibronectin adhesin Pav-type (FIBR). Therefore, strategies should be explored to prevent the emergence of this type of resistance, which, when combined with co-stimulation of antibiotic resistance, could increase the emergence of bacteria and infections resistant to antimicrobial drugs. To avoid resistance generation against AgNPs, the study suggested the combined use of nanoparticles prepared with different metals to prevent the spread of resistant strains (Salas-Orozco et al., 2022).

The transfer of antibiotic resistance genes (ARGs) is considered one of the main ways of spreading resistance, which occurs through horizontal gene transfer (HGT). There are reports that AgNPs enhance the transfer of ARGs via plasmid transmission between bacterial genera. It was determined that both AgNPs and ionic silver, in environmentally relevant and sublethal concentrations, facilitate the transfer of the IncP-α RP4 plasmid produced and supplied by *Escherichia coli* K-12 LE392 to the recipient bacterium *Pseudomonas putida* KT2440. Based on proteomic analyses, genetic sequencing, the potential ecological risks of environmental levels of AgNPs and silver ions in the promotion and dissemination of ARGs by HGT were exposed. Thus, warning against the excessive use of AgNPs in personal care formulations as antimicrobial agents (Lu et al., 2020).

### 6.5 Challenges in the applications of AgNPs: a proteomic view

Among the metallic nanoparticles consumed daily, AgNPs have become the most common due to their antimicrobial effect. A study concerned with the effects of sublethal concentrations of AgNPs on intestinal biofilms investigated the *in vitro* proteomic response of a simulated intestinal biofilm with *E. coli* monospecies to chronic and acute exposures of sublethal concentrations of AgNPs. Of the 1,917 proteins identified, 212 showed different levels of expression. Several pathways were altered, including biofilm formation, bacterial adhesion, response to reactive oxygen species stress, and glucose utilization (Domingo et al., 2019). These findings contribute to a better understanding of the molecular basis of the antibacterial activity of AgNPs, highlighting potential targets for the development of new antimicrobial agents or new therapies that combine classical antimicrobial agents and nanoparticles. Thereby, improving the antimicrobial arsenal against infections caused by multidrug-resistant microorganisms affecting humans and animals (Liu et al., 2023). However, alongside these advancements, it is crucial to consider the potential impact on the natural microbiota (Domingo et al., 2019), as well as the interaction of AgNPs with other cells and biological structures of organisms. Since AgNPs are known to significantly impact living cells, their widespread use raises concerns about both immediate and long-term effects following exposure. For example, a proteomic study with macrophages showed that some functions of these specialized cells, such as lipopolysaccharide-induced cytokine production and nitric oxide, did not return to baseline even 72 h after exposure, showing that some effects of AgNPs persist even after exposure has ceased. This process of recovery of homeostasis after acute exposure to AgNPs involves hundreds of proteins and an enormous energy consumption, 50% higher than normal. The persistence of changes in the proteomic profile may be related to the intracellular silver persistence during the recovery period. Although the concentration of cellular lethality is not reached, post-exposure effects can impact the health status of living organisms (Dalzon et al., 2019). Proteomic analysis has become a valuable tool for identifying new biomarkers with greater sensitivity and specificity, contributing to a better understanding of disease progression, the development of new drugs and combined therapeutic strategies, as well as the improvement of toxicological studies. The identification and quantification of proteins in cells, tissues, or organisms provide insight into the activity of new therapeutics at specialized and personalized molecular levels (Piatek et al., 2023).

## 7 Future perspectives and emerging trends

Silver nanoparticles (AgNPs) have emerged as a promising antimicrobial agent, exhibiting potent activity against a broad spectrum of microorganisms, facilitated by a multifaceted mechanistic approach that enables them to interact with microbial cells in multiple ways. Proteomics has played a crucial role in elucidating the mechanisms of action of AgNPs at the molecular level, revealing that they disrupt cellular processes, including protein synthesis, membrane transport, and cell signaling pathways. These insights have been pivotal in understanding the multifaceted antimicrobial mechanisms of AgNPs (More et al., 2023). The use of AgNPs as antimicrobial agents offers a promising solution to combat the growing threat of antibiotic resistance, a global health concern and their applications in medical devices, wound healing, and water treatment are being explored.

Future perspectives for AgNPs as antimicrobial agents lie in their potential to be combined with other therapeutic agents, such as antibiotics and antioxidants, to enhance their efficacy and reduce toxicity (Chakravarty et al., 2022). Emerging trends include the development of targeted AgNPs that can selectively target specific microorganisms and reduce off-target effects. Additionally, the application of AgNPs in understanding microbial proteomics and identifying novel antimicrobial targets presents a thrilling research avenue, particularly in the face of the escalating global crisis of AMR. As AMR poses a significant threat to the effectiveness of conventional antibiotics against bacterial infections, the WHO’s Global Antimicrobial Resistance and Use Surveillance System (GLASS) has reported alarmingly high resistance rates among common bacterial pathogens, underscoring the urgent need for innovative solutions. Therefore, the potential of AgNPs to revolutionize the field of antimicrobial therapy, and further research is required to fully explore their potential and address the challenges associated with their use.

## Author contributions

AR: Conceptualization, Writing – original draft, Writing – review & editing. JB: Conceptualization, Writing – original draft, Writing – review & editing. MR: Writing – original draft, Writing – review & editing. VT: Writing – review & editing, Writing – original draft. LM: Supervision, Funding acquisition, Writing – review & editing. PL: Supervision, Funding acquisition, Writing – review & editing. AL: Funding acquisition, Supervision, Conceptualization, Resources, Writing – review & editing.
